# Vaccination strategies against wild poliomyelitis in polio-free settings: outbreak risk modelling study and cost-effectiveness analysis

**DOI:** 10.1136/bmjgh-2024-016013

**Published:** 2025-03-22

**Authors:** Megan Auzenbergs, Kaja Abbas, Corey M Peak, Arend Voorman, Mark Jit, Kathleen M O’Reilly

**Affiliations:** 1 Department of Infectious Disease Epidemiology and Dynamics, London School of Hygiene & Tropical Medicine, London, UK; 2 Nagasaki University School of Tropical Medicine and Global Health, Nagasaki, Japan; 3 Institute of Tropical Medicine, Nagasaki University, Nagasaki, Japan; 4 The Gates Foundation, Seattle, Washington, USA; 5 Institute for Disease Modeling, Seattle, Washington, USA

**Keywords:** Immunisation, Health economics, Vaccines, Poliomyelitis, Global Health

## Abstract

The 2021 importation of wild poliovirus serotype 1 (WPV1) into Malawi with subsequent international spread represented the first WPV1 cases in Africa since 2016. Preventing importations and spread of WPV1 is critical and dependent on population immunity provided through routine immunisation (RI) and supplementary immunisation activities (SIAs). We aim to estimate outbreak risk and costs, given the importation of WPV1 for non-endemic countries in the WHO Africa region. We developed a stochastic mathematical model of polio transmission dynamics to evaluate the probability of an outbreak, expected number of poliomyelitis cases, costs and incremental cost-effectiveness ratios under different vaccination strategies. Across variable RI coverage, we explore three key strategies: RI+outbreak SIAs (oSIAs), RI+oSIAs+annual preventative SIAs (pSIAs) and RI+oSIAs+biennial pSIAs. Results are presented in 2023 USD over a 5year- time horizon from the Global Polio Eradication Initiative (GPEI) and health system perspectives. The annual pSIA strategy has the greatest probability of no outbreaks in comparison to other strategies: under our model assumptions, annual pSIAs result in an 80% probability of no outbreaks when RI coverage is ≥50%. The biennial pSIA strategy requires RI coverage ≥65% to achieve an equivalent risk of no outbreaks. The strategy with no pSIAs requires ≥75% RI coverage to achieve an equivalent risk of no outbreaks. For the health system, when RI coverage is between 35% and 60%, both pSIA strategies are cost-saving. For the GPEI, below 65% RI pSIA strategies are cost-effective, but the biennial pSIA strategy incurs higher costs in comparison to annual pSIAs due to more oSIAs required to stop outbreaks. Prioritisation of pSIAs must balance outbreak risk against implementation costs, ideally favouring the smallest manageable outbreak risk compatible with elimination. We infer that there are few short-term risks due to population immunity from RI, but without pSIAs, long-term risks accumulate and can result in outbreaks with the potential for international spread.

**WHAT IS ALREADY KNOWN ON THIS TOPIC**

- Vaccination through both routine immunisation (RI) and supplementary immunisation activities (SIAs) is important for polio eradication. There are proactive preventative SIAs (pSIAs) and reactive outbreak response SIAs, both of which are more costly than RI.
- In 2021–2022, there were importations of wild poliovirus serotype 1 (WPV1) in Africa, a region previously certified polio-free.
- There is a delicate balance between the frequency of costly pSIAs and outbreak risk; however, previous studies do not evaluate cost-effectiveness and outbreak risk, given the potential importation of WPV1 in currently polio-free geographies in Africa.

**WHAT THIS STUDY ADDS**

- Conducting annual pSIAs in areas with low RI coverage is cost-saving and averts more risk of an outbreak following a WPV1 importation than biennial pSIAs or no pSIAs.
- When RI coverage is higher, pSIAs are no longer cost-effective, and the frequency may be reduced with no change in outbreak risk, but it is not until perfect (100%) RI coverage is achieved that there is no risk of an outbreak in the absence of any pSIAs.

**HOW THIS STUDY MIGHT AFFECT RESEARCH, PRACTICE OR POLICY**

- Evaluating the cost-effectiveness of pSIAs for different thresholds of RI coverage is generalisable to many geographies and has policy implications for decision-making.
- The polio eradication strategy includes cessation of the oral polio vaccine; however, to prepare for this cessation, we need to understand outbreak risk in geographies with variable RI coverage.

## Introduction

 In 2019, the African region was certified free from endogenous transmission of wild poliovirus (WPV), with the last clinical case reported in Nigeria in August 2016.^1^ However, in late 2021 and early 2022, Malawi and Mozambique reported WPV serotype 1 (WPV1) cases, respectively, linked to ongoing circulation in Pakistan.^2^ The geographic distribution and genetic linkage of these WPV1 cases suggest missed transmission of an unknown geographic extent.^2^ These WPV1 cases highlight the importance of ensuring high and homogeneous levels of population immunity despite decreasing global incidence and elimination in the African continent.

Poliovirus infection typically initiates in the gut, and approximately one in every 200 infections of serotype 1 may go on to infect the central nervous system and the spinal cord, resulting in a paralytic disease known as poliomyelitis or polio. Since 2016, the recommended routine immunisation (RI) schedule is with the bivalent oral polio vaccine (bOPV) and at least 1 dose of the inactivated polio vaccine (IPV). OPV induces mucosal immunity and protects against infection (and transmission), while IPV only protects against poliomyelitis and does not induce mucosal immunity. OPV is integral to eradication as it prevents infection and transmission. However, variable RI coverage leads to differential population immunity across countries. The genetic instability of the attenuated virus can result in mutations that increase transmissibility and neurovirulence of infections, leading to outbreaks of circulating vaccine-derived poliovirus (cVDPV). Although cVDPVs are not the focus of this work, it is worthwhile to note the current geographic spread of cVDPVs in the African continent when making decisions for polio vaccination programming—a total of 532 cVDPV2 cases were confirmed in 26 countries during January 2023–June 2024.^3^

**Figure 1:**
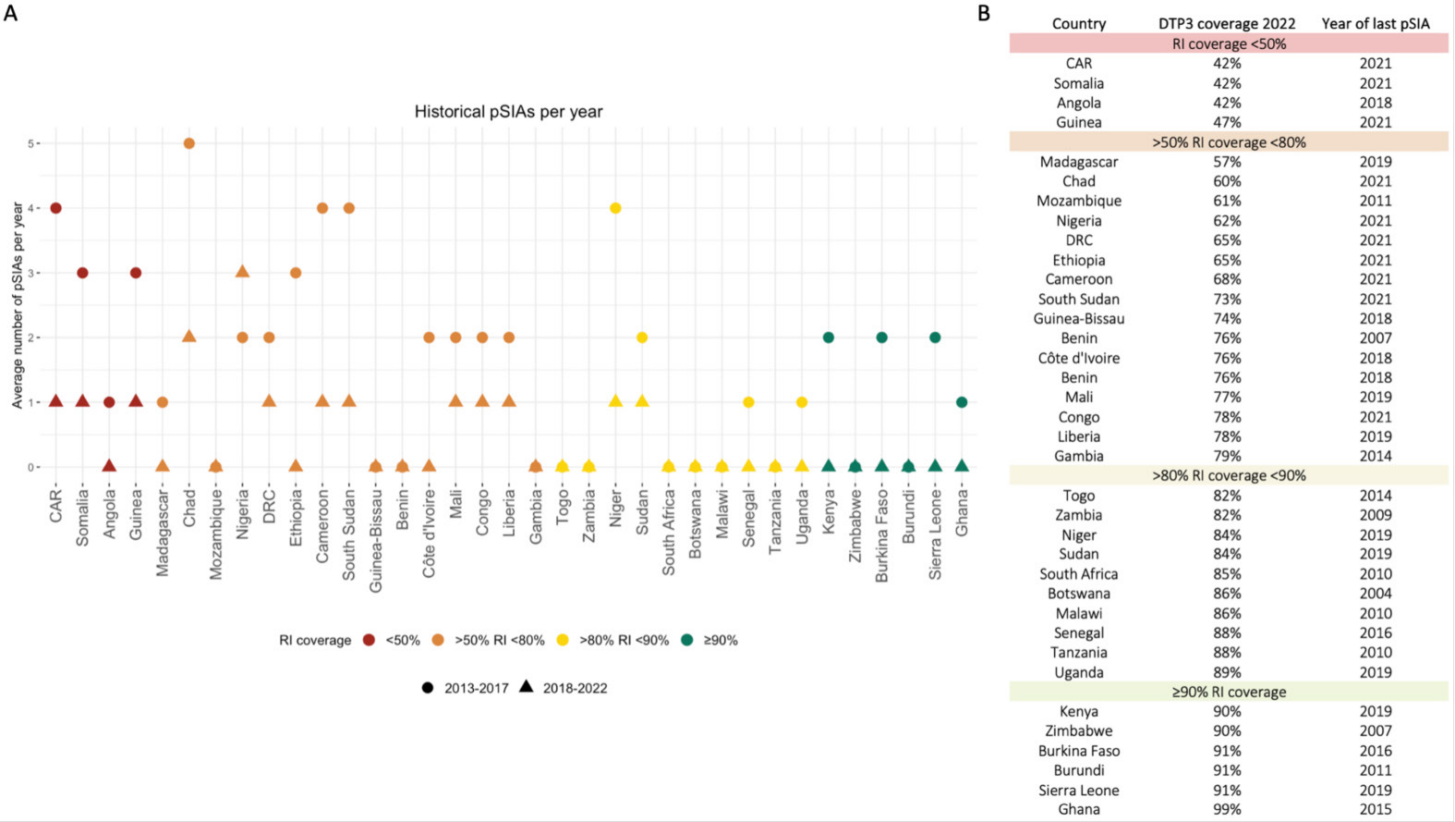
Historical pSIAs and RI coverage in African countries. (A) Mean number of pSIAs per year from 2013 to 2017 and 2018–2022 and (B) year of last pSIA and WHO and UNICEF (WUENIC) estimates of diphtheria tetanus toxoid and pertussis vaccine third dose (DTP3) coverage, an indicator of vaccination via RI as the DTP vaccine is administered concurrently with OPV in the routine immunisation series. Preventative SIAs were defined as either a national or subnational immunisation day (NID, SNID) with bOPV (or trivalent oral polio vaccine (tOPV) pre-2016) and did not occur within 365 days after a WPV1 or VDPV1 detection to distinguish historic pSIAs from oSIAs. Any SIAs that occurred within 365 days of a WPV1 or VDPV1 outbreak were not included in the pSIA count. Country selection represents low-, lower-middle- and upper-middle-income sub-Saharan African countries. bOPV, bivalent oral polio vaccine; CAR, Central African Republic; DRC, Democratic Republic of the Congo; OPV, oral polio vaccine; oSIAs, outbreak supplementary immunisation activities; pSIAs, preventative supplementary immunisation activities; RI, routine immunisation.

The Global Polio Eradication Initiative (GPEI) is responsible for the coordination of activities to support polio eradication. The activities include surveillance for acute flaccid paralysis (AFP) which includes poliomyelitis and other infectious and non-infectious causes, environmental surveillance for poliovirus and providing polio vaccinations through both supplementary immunisation activities (SIAs) and RI (through the Expanded Programme for Immunisation). SIAs typically aim to vaccinate all children under 5 years old, including those hard-to-reach children otherwise missed by RI. Despite an annual expenditure of around US$1 billion, decision makers within polio eradication often must make complex decisions in allocating resources amid decreases in the global budget.^4^ Alongside, the frequency of preventative SIAs (pSIAs) has decreased in almost all countries in Africa since 2017 (figure 1).^5^

The GPEI annual budget consists of contributions from donors and is used to support the GPEI’s objectives. This budget is divided into pSIAs and outbreak response and additional budget lines (not considered further in this study). Outbreak response includes outbreak response SIAs (oSIAs), while pSIAs are planned to prevent outbreaks in polio-free settings and raise population immunity in at-risk areas to stop transmission. Operationally, pSIAs and oSIAs differ both in the target populations for vaccination as well as the funding and planning for activities – oSIAs must be implemented soon after outbreak detection and require more resources for rapid mobilisation. pSIAs are planned well ahead of implementation and are less costly because logistics do not require rapid mobilisation, but as their need is not always acute, this can result in deprioritisation.

Since the World Health Assembly’s 1988 resolution to eradicate polio by the year 2000, economic analyses have informed strategies to progress towards this goal.^6^,^8^ However, few studies distinguish between pSIAs and oSIAs, which is important because they have different strategic goals and funding approaches. Furthermore, of the economic analyses that include modelling of different vaccination strategies, several assume eradication will have already occurred,^7 9^ include limited geographies^7 10^ or model populations where WPV1 is endemic.^11^ A recent economic analysis that considers the cost-effectiveness of operational decisions for pSIAs and oSIAs in a hypothetical setting does not consider importations of WPV1.^12^ Therefore, we provide a modelling approach for low-and-middle-income countries (LMICs) in sub-Saharan Africa to compare strategies of differing frequencies of pSIAs to identify at what levels of RI the risks of outbreaks and polio cases may outweigh the associated costs of implementing pSIAs, given the risk of WPV1 importations. Our work differs from past research because it (i) considers the imminent risk of WPV1 importations into currently polio-free geographies in Africa, (ii) evaluates the benefits and trade-offs (outbreak risk and cost-effectiveness) of pSIAs in comparison to a baseline scenario relying only on oSIAs and RI and (iii) estimates the cost-effectiveness of varying frequencies of pSIAs. By modelling a hypothetical polio-free LMIC in sub-Saharan Africa, we aim to present relevant results for a range of geographies with different levels of RI coverage and variable schedules for historical pSIAs. This is an evidence gap identified by stakeholders involved in OPV cessation planning that is important to address as we approach the final stages of WPV1 transmission.^13^

## Methods

We evaluated different vaccination strategies for a hypothetical population of 8 million children under 5 years of age, reflecting a mean population size across 25 LMICs in sub-Saharan Africa (online supplemental appendix p 7). Model outputs from each strategy include probability of an outbreak, estimated cases of paralytic poliomyelitis and vaccine-associated paralytic polio (VAPP), number of outbreaks and disability-adjusted life years (DALYs). The Consolidated Health Economic Evaluation Reporting Standards 2022 reporting guidance was used in the development of this analysis,^14^ online supplemental appendix pp 25 and 26.

### Vaccination strategies

We explored three vaccination strategies (table 1). We assume that vaccination via RI follows a sequential immunisation schedule that includes three doses of bOPV given orally and 1 dose IPV administered intramuscularly or subcutaneously.

**Table 1:** Polio vaccination strategies

Vaccination strategy	RI coverage levels modelled	oSIA% of target population vaccinated	pSIA% of target population vaccinated	pSIA frequency	R_0_	WPV1 importations
Baseline strategy	25%–100% in 5% increments	25%	No pSIAs	No pSIAs	3	Two per year
Annual pSIA strategy	25%–100% in 5% increments	25%	25%	Annual		
Biennial pSIA strategy	25%–100% in 5% increments	25%	25%	Every 2 years		

The target population for SIAs is children missed by RI. For example, both pSIAs and oSIAs vaccinate 25% of the population of children missed by RI.

An outbreak response was only conducted if a simulation had at least 1 case of paralytic polio. Additional assumptions for R_0_, SIA target populations and importation rate are explored in sensitivity analyses (online supplemental appendix pp 22–22).

oSIAoutbreak response supplementary immunisation activitypSIApreventative supplementary immunisation activityRIRoutine Immunisation

In all strategies, we define an outbreak as at least one case of paralytic polio. An oSIA is conducted in all simulations where at least one case was detected within any 90-day interval, in line with standard operating procedures.^15^ oSIAs continue until all cases are stopped over the 5 year time horizon. We do not account for case detections through environmental surveillance in this analysis. We assume that all SIAs reach 25% of children missed by RI, as prior evidence suggests that SIA coverage varies across locations and analysis with higher coverage assumptions for zero-dose children resulted in unrealistically high population immunity when compared with empirical data.^16^ A sensitivity analysis of different SIA assumptions is on online supplemental appendix p 20.

### Model structure

We developed a stochastic SIR model to simulate polio transmission dynamics, whereby infectious individuals develop either asymptomatic or symptomatic infection, both of which are assumed to be infectious. We specify vaccine-induced immunity based on OPV and IPV doses. In the model, children under the age of 5 years are either susceptible, fully vaccinated and protected from poliovirus infection, or have received an incomplete vaccination series (less than three bOPV doses+one IPV dose). Each subsequent dose of vaccine corresponds to additional protection and an opportunity for a child to seroconvert and be considered fully protected from infection (online supplemental appendix pp 3–5).

While bOPV also has protective effects against poliovirus serotype 3, we only consider the vaccine’s protective effects for serotype one for this analysis due to the greater risk of WPV1 given recent importations. Outbreaks of cVDPV serotype 2 require alternative vaccines and assumptions and are therefore beyond the scope of this analysis. cVDPV serotypes 1 and 3 are also outside of the scope of this analysis due to the specific mechanisms of emergence.

### Model assumptions

The modelled time horizon is 5 years, in line with the current GPEI strategic plan 2022–2026 where a central aim is to interrupt all WPV transmission in the coming years.^17^ R_0_ is the basic reproductive number and estimates the expected number of secondary poliovirus infections in an immunologically naïve population. We have used an R_0_ of 3, supported by data-driven work exploring variable R_0_ values in a non-endemic setting in Africa for children under 5 years of age,^18^ and higher R_0_ assumptions were explored in sensitivity analysis (online supplemental appendix p 21). The proportion of children vaccinated with one dose of IPV is assumed to be equal to the third dose of bOPV RI coverage, in line with the joint assessment of immunisation coverage by the WHO and UNICEF (WUENIC) data^19^ (online supplemental appendix p 7).

We assume a randomly mixed population of children under 5 with no heterogeneity in the probability of a child being vaccinated in an SIA or in transmission of poliovirus. Data shows a low mean age of wild poliomyelitis infections, with under-5s accounting for more than 80% of cases in non-endemic settings.^20^ Older children and adults are thought to play a minor role in WPV transmission (with a few notable exceptions);^16 20^ therefore, we focus only on children under 5 for this analysis. Simulations were run for 50 years before virus introduction, allowing for historical pSIAs, then one infection was introduced into the population at the start of the simulation, and further WPV1 importations were assumed to occur at a Poisson-distributed rate of two importations per year. Different importation rates and seasonality are addressed in a sensitivity analysis (online supplemental appendix p 22). The models were repeated for 10 000 stochastic simulations and run using the R package SimInf in R V.4.2.2.^21^

### Outbreak probability

The probability of an outbreak was calculated using the proportion of stochastic simulations that resulted in at least one paralytic polio case (ie, a polio AFP case) following the importation of WPV1. This definition is not directly comparable to the WHO criteria for elimination status^22^ but is useful for understanding outbreak risk. For example, the WHO criteria for elimination refer to the reduction to zero of the incidence of infection caused by a poliovirus in a defined area.^22^

### Disability-adjusted life years

DALYs were calculated assuming that in LMICs, the mean discounted lifetime DALYs associated with one paralytic poliomyelitis case, with no age-weighting, is 14 DALYs per paralytic case,^5^ assuming that one in 200 infections leads to irreversible paralysis and among those paralysed, 5%–10% die when respiratory muscles become paralysed,^23^ and long-term mortality is approximately 20% higher in paralytic polio cases than the general population.^24^ The proportional contribution of years lost to disability (YLD) and years of life lost due to premature mortality (YLL), assuming a mean of 14 DALYs per case, is 60% YLDs in addition to 40% YLLs per case.^25^ After the importation of infection, we assume no further international transmission during outbreaks for calculations of DALYs.

### Health and economic outcomes

Incremental costs and DALYs averted were used to estimate incremental cost-effectiveness ratios (ICERs) under each pSIA strategy, calculated as follows:

$$ICER=\frac{\left(costs\;of\;pSIA\;strategy-costs\;of\;baseline\;strategy\right)}{\left(DALYs\;averted\;by\;pSIA\;strategy\right)}$$

We compare the ICER to three thresholds determined by Pichon-Riviere *et al* 2023^26^ representing the lowest, median and highest cost-effectiveness thresholds among the sub-Saharan African countries used in the sample size calculation (online supplemental appendix p 7). We used a 3% discount rate for costs and 0% for health with no age-weighting,^27^ with other discounting assumptions explored in the online supplemental appendix p 16. We do not include indirect costs of vaccination, such as opportunity costs of time spent for vaccination.

### Perspectives

Incremental costs are analysed from both the GPEI and health system perspectives and a combined perspective for both the health system and GPEI. The GPEI perspective is valuable for strategic planning and future programming as well as for domestic health systems in their overall polio programming activities. For discounting, costs are calculated annually for each model simulation and then aggregated over all simulations and the 5-year time horizon.

The total costs for the health system perspective are calculated as follows:

*(cost per AFP case×AFPcases) + (cost per VAPP case×VAPPcases) + (RI coverage × (newborns eligible for bOPV vaccination×total doses received per child) × (cost per dose of bOPV+RI delivery cost per dose of bOPV) × (1 + (bOPV wastage rate for RI / (1 – bOPV wastage rate for RI)))*

The total costs for the GPEI perspective are calculated as follows:

*(SIA coverage × (target population* ^*†*^ *×numberof pSIAs) × (cost per dose of bOPV+pSIA delivery cost per dose of bOPV) × (1+(bOPV wastage rate for SIAs / (1 – bOPV wastage rate for SIAs))) + (SIA coverage × (target population* ^*†*^ *×numberof oSIAs) × (cost per dose of bOPV+oSIA delivery cost per dose of bOPV) × (1 + (bOPV wastage rate for SIAs / (1 – bOPV wastage rate for SIAs))) + (RIcoverage × (newborns eligible for IPV vaccination) × (cost per dose of IPV+RI delivery cost per dose of IPV) × (1 + (bOPV wastage rate for RI / (1 – bOPV wastage rate for RI)))*

^†^Target population for pSIAs and oSIAs refers to all children under 5 years of age.

### Vaccine costs

Gavi, The Vaccine Alliance, supports the world's poorest countries by co-financing vaccines. Vaccine costs per dose for bOPV and IPV in Gavi-supported countries were obtained from the latest UNICEF update in 2023 USD, with a mean cost of $0.18 and $2.00, respectively,^28 29^ and costs associated with RI (administration, procurement and storage) were obtained from previous research,^30^ online supplemental appendix p 23. All costs have been adjusted to 2023 USD. The main analysis assumes 10% wastage for OPV in SIAs, 13% wastage for OPV in RI and 13% for IPV.^31 32^ Further wastage assumptions are in the online supplemental appendix p 17.

### SIA data and costs

The Polio Information System was used to obtain SIA data from 2013 to 2022, and further analysis was done to distinguish pSIAs from oSIAs (online supplemental appendix p 1). The cost per child for pSIAs and oSIAs was obtained from GPEI data (online supplemental appendix p 23) and ranged from USD2023 $0.28–$1.12 for pSIAs and USD2023 $0.22–$2.79 for oSIAs. We assume oSIAs cost twice the cost of a pSIA and explore a range of proportional costs between pSIAs and oSIAs (online supplemental appendix pp 18–19). The stochasticity of outbreaks, which affects total estimated costs, is variable and contributes to the variability in expected costs across all strategies (online supplemental appendix p 10).

### Adverse events

The expected risk of adverse events, such as VAPP in countries using OPV is one case of VAPP per 0.9 million doses of bOPV administered and declines with subsequent doses.^33^ A VAPP case was considered equivalent to a case of wild-acquired paralytic polio for calculation of the expected costs of VAPP and DALYs.

### Patient and public involvement

Patients were not involved in this research.

## Results

**Figure 2:**
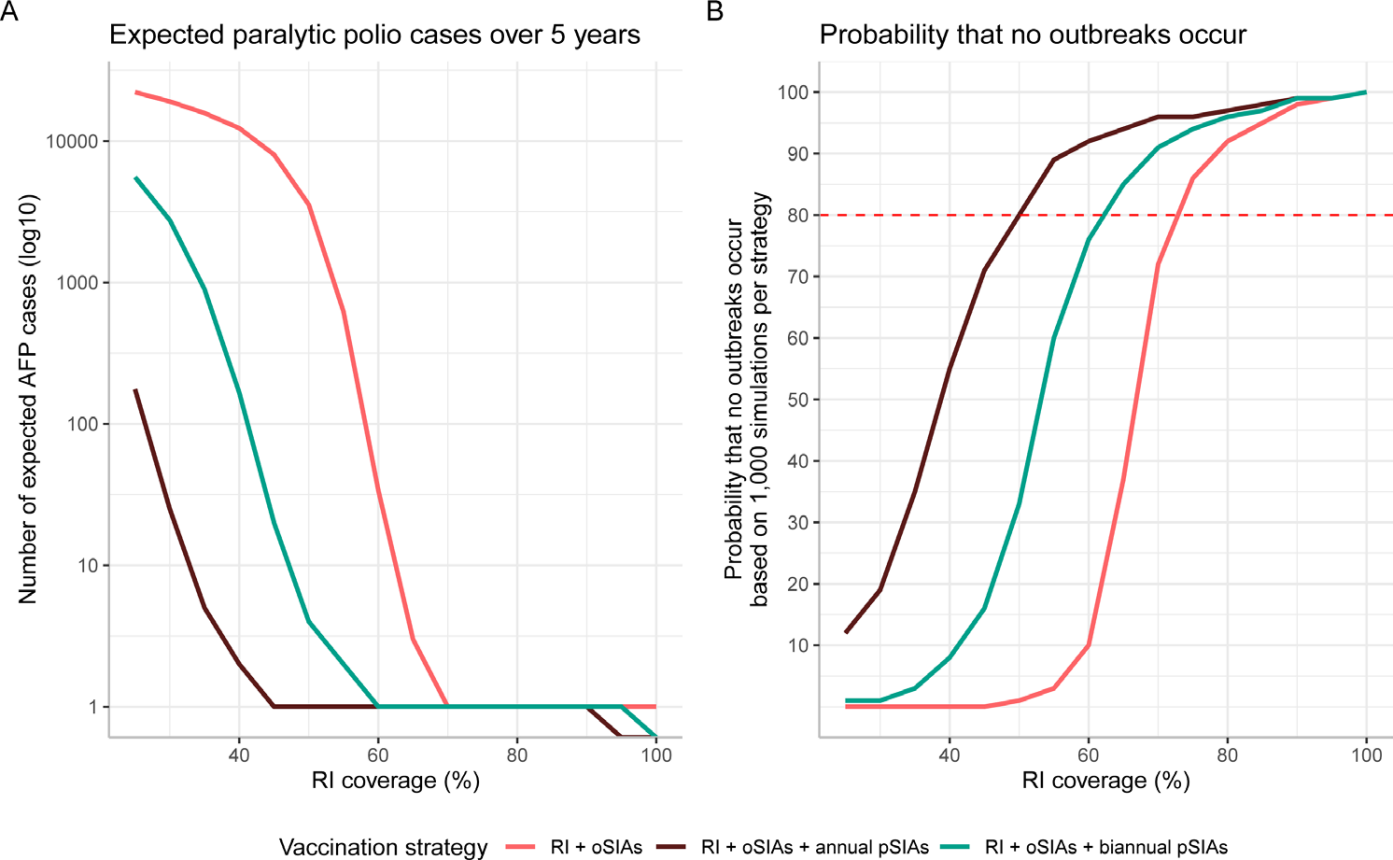
Estimated number of paralytic polio cases and the probability that no outbreaks occur over 5 years. (A) Number of expected paralytic polio cases (presenting as a polio AFP case). The solid line represents the mean estimate of 10 000 simulations and (B) the probability of no outbreaks occurring across all vaccination strategies. Outbreak probability was based on 10 000 simulations per vaccination strategy. The red dashed line corresponds to an 80% probability that no outbreaks occur. AFP, acute flaccid paralysis; oSIAs, outbreak supplementary immunisation activities; pSIAs, preventative supplementary immunisation activities; RI, routine immunisation.

Across all simulations, the mean expected number of WPV1 cases over 5 years is greatest in the baseline strategy and least in the annual pSIA strategy (figure 2A). The annual pSIA strategy is the strategy under which the fewest number of outbreaks occur across all RI coverage levels. Under the base case assumptions (including R_0_ and the proportion of zero-dose children reached by SIAs), annual pSIAs achieve and maintain >80% probability of no outbreaks when baseline RI coverage is 50% (figure 2B). The biennial pSIA strategy achieves >80% probability of no outbreaks when RI is above 65%, and the baseline strategy requires ≥75% RI coverage to achieve >80% probability of no outbreaks.

**Figure 3:**
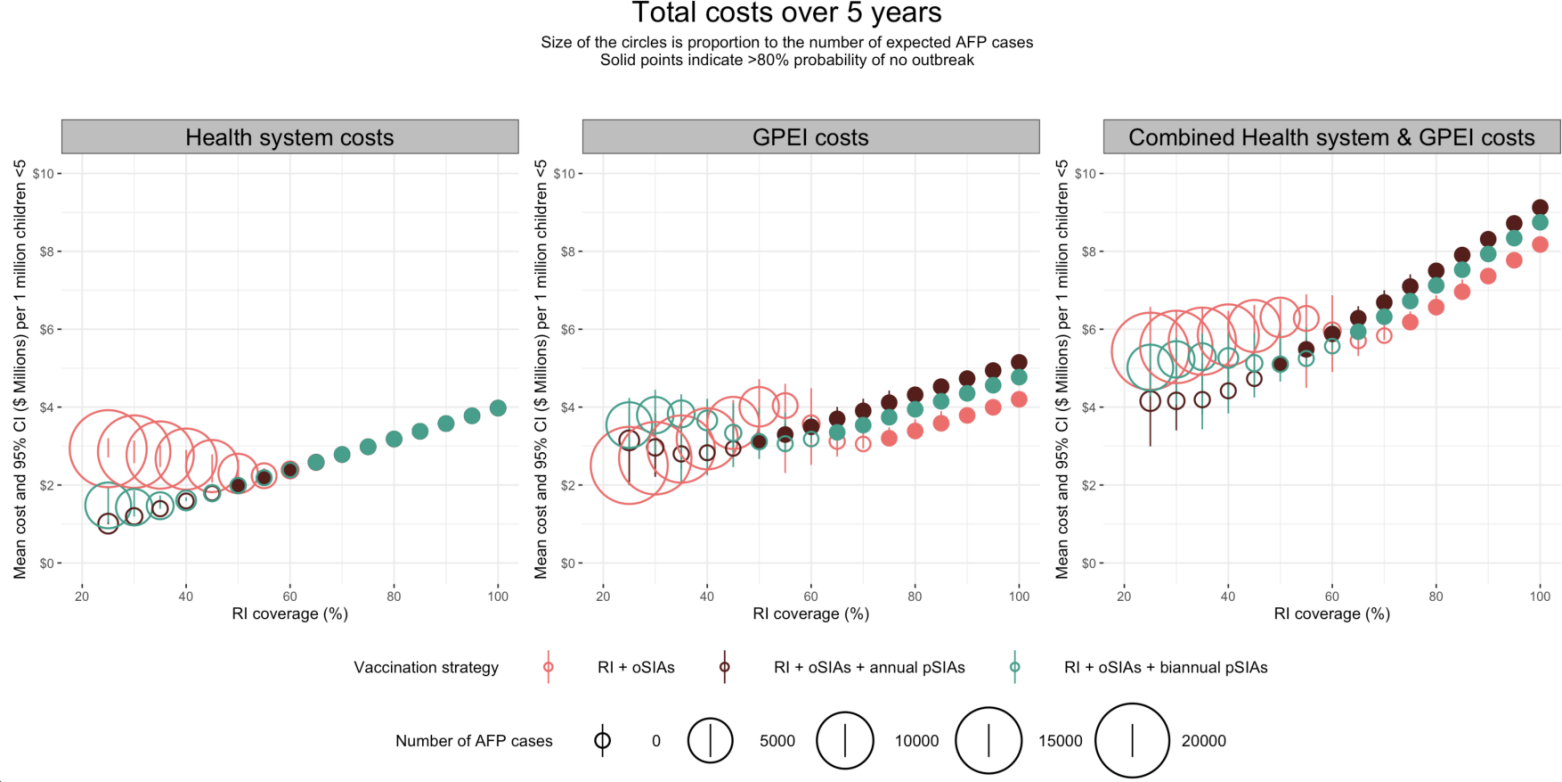
Health system and GPEI estimated total costs over 5 years. The size of the circle is proportional to the mean number of expected paralytic polio cases across all model simulations. The solid circles correspond to >80% probability that the strategy had no outbreaks over a 5-year period. AFP, acute flaccid paralysis; GPEI, Global Polio Eradication Initiative; oSIAs, outbreak supplementary immunisation activities; pSIAs, preventative supplementary immunisation activities; RI, routine immunisation.

The annual pSIA strategy had the greatest expected VAPP cases over 5 years, since it was the strategy with the greatest number of vaccine doses administered and resulted in the fewest expected WPV1 cases over 5 years. Estimated costs are shown in figure 3 and online supplemental appendix pp 12–14. Calculating herd immunity as (1–1/R_0_), when RI coverage is below 66.6%, the point when herd immunity is achieved in this simple homogenously mixed model, total costs from all perspectives are highest in the baseline strategy. Above the herd immunity threshold, costs for the health system perspective are comparable across all strategies, due to fewer paralytic cases with increasing RI coverage. From the GPEI perspective, from 25%–40% RI coverage, costs are highest in the biennial pSIA strategy, driven by more oSIAs than the annual pSIA strategy. When RI coverage is 45%–60%, the baseline strategy has the greatest costs due to a greater number of oSIAs required to stop outbreaks. When RI coverage exceeds the herd immunity threshold, costs are highest in the annual pSIA strategy due to the high costs associated with annual campaigns and an increase in the number of VAPP cases with increasing RI coverage. From the combined health system and GPEI perspective, below the herd immunity threshold, costs are highest in the baseline strategy, due to the large number of AFP cases. Above the herd immunity threshold, the annual pSIA strategy becomes the costliest strategy.

**Figure 4:**
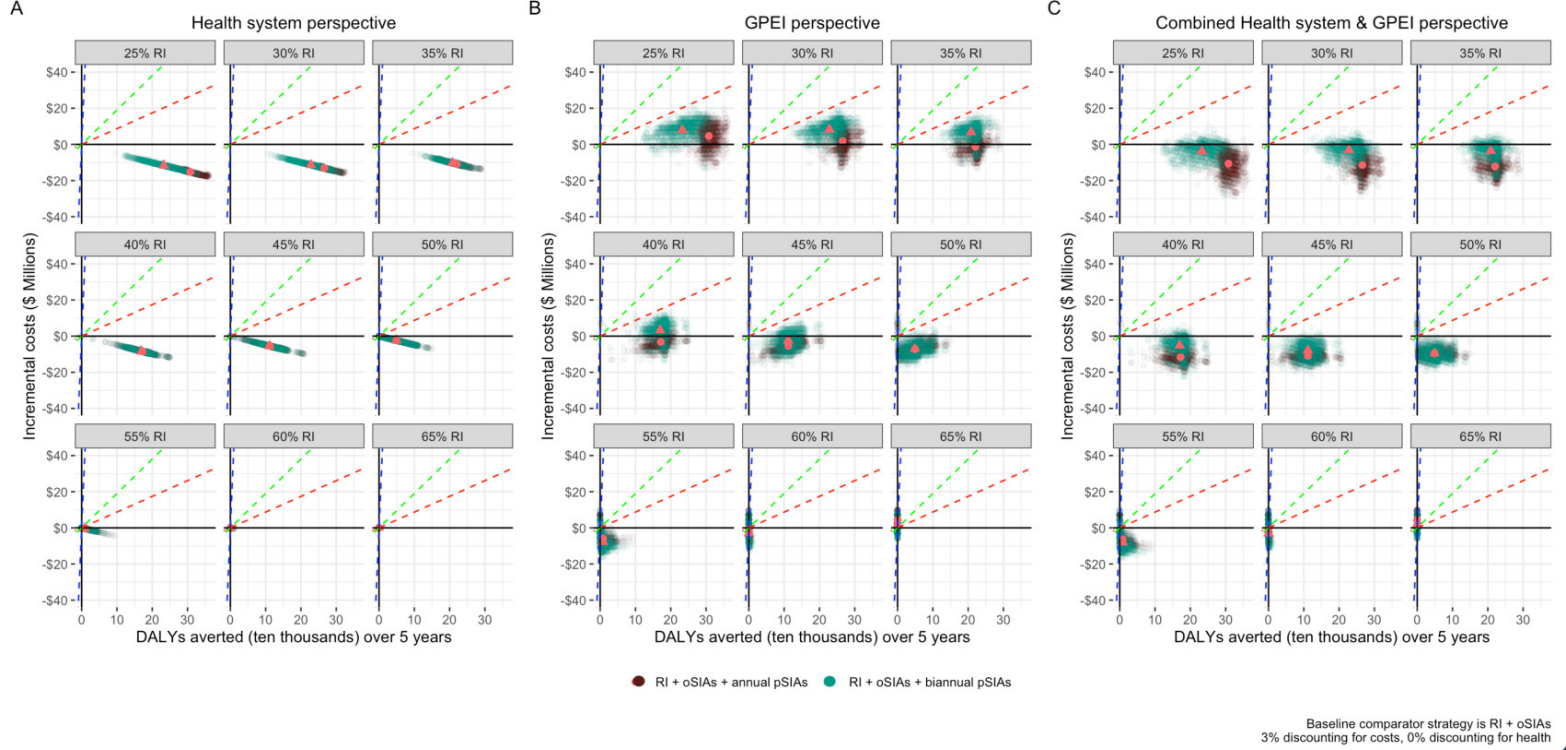
Cost-effectiveness planes for the annual and biennial pSIA vaccination strategies. Incremental costs and DALYs averted under the annual pSIA (RI+oSIAs+annual pSIAs) and biennial pSIA (RI+oSIAs+Biennial pSIAs) strategies are compared with the baseline strategy (RI+oSIAs). The pink circle is the mean estimate for the annual pSIA strategy, and the pink triangle is the mean estimate for the biennial pSIA strategy. Each individual model simulation is represented as a single dot. The dashed lines represent three cost-effectiveness thresholds (representing the lowest (red=Democratic Republic of the Congo), median (green=Benin) and highest (blue=South Africa) country thresholds) among low-, lower-middle- and upper-middle-income sub-Saharan African countries. DALYs, disability-adjusted life years; GPEI, Global Polio Eradication Initiative; oSIAs, outbreak supplementary immunisation activities; pSIAs, preventative supplementary immunisation activities; RI, routine immunisation.

For the health system (figure 4A and table 2), when RI coverage is below 66.6%, both pSIA strategies are cost-saving at the country-level upper, median and lower bounds (see online supplemental appendix p 24 for further explanation of the quadrants of a cost-effectiveness plane). Above the herd immunity threshold, no DALYs are averted by either pSIA strategy; instead, more DALYs are incurred with pSIAs due to VAPP, hence the negative ICERs. From the GPEI perspective, when RI is 25%–30%, the ICERs for annual pSIAs are USD$15 and USD$7 per DALY averted, but then the strategy becomes cost-saving between 30% and 60% RI (figure 4B and table 3). For biennial SIAs, the strategy is more costly when RI coverage is 25%–40% and cost-saving from 45%–60% RI coverage. When the health system and GPEI perspectives are combined, both pSIA strategies are cost-saving when RI coverage is below the herd immunity threshold (figure 4C and table 4). When RI coverage approaches 66.6%, the point when herd immunity is achieved, the ICERs for all perspectives (tables2^4^) are negative for both annual and biennial pSIAs due to increased VAPP cases in comparison to the baseline strategy. However, even if the pSIA strategies are not cost-effective at >66.6% RI coverage and present challenges for VAPP, both pSIA strategies continue to avert outbreaks as RI coverage increases, which is important as a single outbreak under any vaccination strategy has implications for global polio eradication (online supplemental appendix p 11).

**Table 2:** Health system perspective—DALYs averted and differential costs between each pSIA strategy and the baseline strategy

RI coverage	pSIA strategy	DALYs averted	Cost difference	ICER	Commentary
25	Annual pSIAs	307 907	−15311684	−50	Cost saving
30	Annual pSIAs	265 881	−11656192	−50	
35	Annual pSIAs	220 350	−13204856	−50	
40	Annual pSIAs	171 969	−11384118	−50	
45	Annual pSIAs	111 684	−10904822	−49	
50	Annual pSIAs	49 157	−10327265	−50	
55	Annual pSIAs	8172	−8492658	−49	
60	Annual pSIAs	185	−8392864	−49	
65	Annual pSIAs	−237	−5487803	−49	No DALYs averted; instead, more DALYs incurred with pSIAs due to VAPP, hence the negative ICER
70	Annual pSIAs	−287	−5480087	−49	
75	Annual pSIAs	−309	−2392834	−49	
80	Annual pSIAs	−327	−2394881	−49	
85	Annual pSIAs	−345	−392734	−48	
90	Annual pSIAs	−362	−396638	−48	
95	Annual pSIAs	−379	−8804	−48	
100	Annual pSIAs	−397	−13386	−47	
25	Biennial pSIAs	232 850	11 382	−48	Cost saving
30	Biennial pSIAs	228 148	6353	−49	
35	Biennial pSIAs	208 311	13 775	−48	
40	Biennial pSIAs	169 876	8351	−49	
45	Biennial pSIAs	111 523	14 832	−48	
50	Biennial pSIAs	49 200	9052	−49	
55	Biennial pSIAs	8255	15 716	−48	
60	Biennial pSIAs	283	9594	−48	
65	Biennial pSIAs	−130	16 560	−48	No DALYs averted; instead, more DALYs incurred with pSIAs due to VAPP, hence the negative ICER
70	Biennial pSIAs	−172	10 095	−49	
75	Biennial pSIAs	−186	17 387	−48	
80	Biennial pSIAs	−198	10 584	−49	
85	Biennial pSIAs	−208	18 204	−48	
90	Biennial pSIAs	−218	11 065	−49	
95	Biennial pSIAs	−228	19 053	−48	
100	Biennial pSIAs	−238	11 569	−49	

Interpretation of the ICERs is provided in the commentary column. Negative ICERs are usually cost -saving, but>65%,but >65% negative ICERs are due to VAPP.

DALYsdisability-adjusted life yearsICERsincremental cost-effectiveness ratiospSIAspreventative supplementary immunisation activitiesRIroutine immunisationVAPPvaccine-associated paralytic polio

**Table 3:** GPEI perspective—DALYs averted and differential costs between each pSIA strategy and the baseline strategy

RI coverage	pSIA strategy	DALYs averted	Cost difference	ICER	Commentary
25	Annual pSIAs	307 907	4 668 615	15	More costly
30	Annual pSIAs	265 881	1 792 608	7	
35	Annual pSIAs	220 350	−1312169	-6	Cost saving
40	Annual pSIAs	171 969	−3140650	−18	
45	Annual pSIAs	111 684	−5392966	−48	
50	Annual pSIAs	49 157	−7111413	−145	
55	Annual pSIAs	8172	−5878933	−719	
60	Annual pSIAs	185	−681367	−3683	
65	Annual pSIAs	−237	4 331 306	−18276	No DALYs averted; instead, more DALYs incurred with pSIAs due to VAPP, hence the negative ICER
70	Annual pSIAs	−287	6 416 874	−22358	
75	Annual pSIAs	−309	6 888 407	−22293	
80	Annual pSIAs	−327	7 022 112	−21474	
85	Annual pSIAs	−345	7 095 951	−20568	
90	Annual pSIAs	−362	7 127 935	−19690	
95	Annual pSIAs	−379	7 127 872	−18807	
100	Annual pSIAs	−397	7 157 937	−18030	
25	Biennial pSIAs	232 850	7 945 495	34	More costly
30	Biennial pSIAs	228 148	8 352 947	37	
35	Biennial pSIAs	208 311	6 679 133	32	
40	Biennial pSIAs	169 876	3 259 477	19	
45	Biennial pSIAs	111 523	−2327649	−21	Cost saving
50	Biennial pSIAs	49 200	−7150458	−145	
55	Biennial pSIAs	8255	−7643058	−926	
60	Biennial pSIAs	283	−3049671	−10776	
65	Biennial pSIAs	−130	1 713 497	−13181	No DALYs averted; instead, more DALYs incurred with pSIAs due to VAPP, hence the negative ICER
70	Biennial pSIAs	−172	3 650 380	−21223	
75	Biennial pSIAs	−186	4 067 384	−21868	
80	Biennial pSIAs	−198	4 203 660	−21231	
85	Biennial pSIAs	−208	4 252 305	−20444	
90	Biennial pSIAs	−218	4 277 093	−19620	
95	Biennial pSIAs	−228	4 274 782	−18749	
100	Biennial pSIAs	−238	4 294 966	−18046	

Interpretation of the ICERs is provided in the commentary column. When RI coverage is 25% and 30% for the annual pSIA strategy, the strategies are more costly than the baseline because the baseline strategy results in explosive outbreaks that deplete susceptibles, resulting in the pSIA strategy requiring more total poSIAs throughout the time horizon.

DALYsdisability-adjusted life yearsGPEIGlobal Polio Eradication InitiativeICERsincremental cost-effectiveness ratiospSIAspreventative supplementary immunisation activitiesRIroutine immunisationVAPPvaccine-associated paralytic polio

**Table 4:** Combined health system and GPEI perspective—DALYs averted and differential costs between each pSIA strategy and the baseline strategy

RI coverage	pSIA strategy	DALYs averted	Cost difference	ICER	Commentary
25	Annual pSIAs	307 907	−10643069	−35	Cost saving
30	Annual pSIAs	265 881	−11412248	−43	
35	Annual pSIAs	220 350	−12216992	−55	
40	Annual pSIAs	171 969	−11633308	−68	
45	Annual pSIAs	111 684	−10880769	−97	
50	Annual pSIAs	49 157	−9504247	−193	
55	Annual pSIAs	8172	−6271667	−767	
60	Annual pSIAs	185	−690171	−3731	
65	Annual pSIAs	−237	4 342 688	−18324	No DALYs averted; instead, more DALYs incurred with pSIAs due to VAPP, hence the negative ICER
70	Annual pSIAs	−287	6 430 649	−22406	
75	Annual pSIAs	−309	6 903 240	−22341	
80	Annual pSIAs	−327	7 037 828	−21522	
85	Annual pSIAs	−345	7 112 511	−20616	
90	Annual pSIAs	−362	7 145 322	−19738	
95	Annual pSIAs	−379	7 146 075	−18855	
100	Annual pSIAs	−397	7 176 991	−18078	
25	Biennial pSIAs	232 850	−3710697	−16	Cost saving
30	Biennial pSIAs	228 148	−3031171	−13	
35	Biennial pSIAs	208 311	−3648131	−18	
40	Biennial pSIAs	169 876	−5133387	−30	
45	Biennial pSIAs	111 523	−7807735	−70	
50	Biennial pSIAs	49 200	−9545339	−194	
55	Biennial pSIAs	8255	−8039696	−974	
60	Biennial pSIAs	283	−3063057	−10824	
65	Biennial pSIAs	−130	1 719 850	−13230	No DALYs averted; instead, more DALYs incurred with pSIAs due to VAPP, hence the negative ICER
70	Biennial pSIAs	−172	3 658 730	−21272	
75	Biennial pSIAs	−186	4 076 436	−21916	
80	Biennial pSIAs	−198	4 213 253	−21279	
85	Biennial pSIAs	−208	4 262 400	−20492	
90	Biennial pSIAs	−218	4 287 677	−19668	
95	Biennial pSIAs	−228	4 285 847	−18798	
100	Biennial pSIAs	−238	4 306 535	−18095	

Interpretation of the ICERs is provided in the commentary column.

DALYsdisability-adjusted life yearsGPEIGlobal Polio Eradication InitiativeICERsincremental cost-effectiveness ratiospSIAspreventative supplementary immunisation activitiesRIroutine immunisationVAPPvaccine-associated paralytic polio

Table 5 outlines the implications for decision-making. When RI coverage falls below 50%, the annual pSIA strategy averts many cases, so removal of pSIAs entirely would create substantial risk. Countries with 50%–90% RI coverage have a higher probability of no outbreaks occurring. However, the risk of an outbreak is not removed entirely until the probability of no outbreaks reaches 100% (when WPV1 transmission is interrupted globally). All strategies require >95% coverage for a 100% probability that no outbreaks occur; however, above 80% RI coverage, outbreak probability is low and, if an outbreak does occur, the expected number of cases is low.

**Table 5:** Policy implications of polio vaccination strategies

RI coverage	Estimated risk with annual pSIAs‡	Estimated risk with biennial pSIAs‡	Estimated risk relying on oSIAs only‡	Outbreaks averted by annual pSIAs	Outbreaks averted by biennial pSIAs	Implications for decision-making
	Mean‡ Polio cases if an outbreak occurs (95% CI)(Probability no outbreaks occur, from 10 000 simulations)			Median (IQR)		
35%	3 (3 to 4)(35%)	401 (368 to 434)(3%)	9191 (8834 to 9547)(0%)	3 (2–4)	−1 (−2–0)	pSIA removal would have high risks and consequences
50%	1 (1 to 1)(80%)	3 (3 to 3)(33%)	1611 (2526 to 1697)(1%)	6 (5–6)	5 (3–6)	Removal of pSIAs altogether could lead to a high risk of outbreaks in subsequent years
55%	1 (1 to 1)(89%)	2 (1 to 2)(60%)	289 (263 to 315)(3%)	5 (5–6)	5 (4–6)	
60%	1 (1 to 1)(92%)	1 (1 to 1)(76%)	20 (17 to 23)(11%)	4 (2–4)	3 (2–4)	
65%	1 (1 to 1)(94%)	1 (1 to 1)(85%)	3 (2 to 3)(37%)	1 (0–2)	1 (0–2)	
70%	1 (1 to 1)(96%)	1 (1 to 1)(91%)	1 (1 to 1)(72%)	0 (0–1)	0 (0–1)	Reducing the frequency of pSIAs could still maintain a low risk of large outbreaks
80%	1 (1 to 1)(97%)	1 (1 to 1)(96%)	1 (1 to 1)(92%)	NA	NA	
90%	1 (1 to 1)(99%)	1 (1 to 1)(99%)	1 (1 to 1)(98%)	NA	NA	
100%	0 (0 to 0)(100%)	0 (0 to 0)(100%)	1 (1 to 1)(100%)	NA	NA	Even if pSIAs are removed, there is low to no risk of outbreaks

Expected paralytic polio cases are conditional means‡ amongstamong simulations that resulted in at least one case. DALYs and outbreaks averted are mean and median values across all model simulations, respectively. The probability of no outbreaks occurring is obtained from the proportion of model simulations (out of 10 000 simulations) that resulted in zero paralytic cases. For outbreaks averted, the comparator is the baseline strategy with no pSIAs. The raw data used to create this table, alongside data for additional RI coverage levels, isare in the online supplemental appendix pp 12–14Appendix pp .

DALYsdisability-adjusted life yearsoSIAsoutbreak supplementary immunisation activitiespSIAspreventative supplementary immunisation activitiesRIroutine immunisation

## Discussion

The key messages of our study include: (i) with higher RI, the probability of outbreaks reduces considerably—under our model assumptions, outbreak size and risk are minimal when RI is above 66.6%; (ii) pSIAs of any frequency avert DALYs and are cost-saving for the combined GPEI and health system perspective below 66.6% RI; (iii) a strategy with only RI and oSIAs implicitly accepts some level of outbreak risk, but if RI is above 70%–80%, the risk of outbreaks is considerably less than in other settings where RI is below 70%, which may be a feasible and cost-effective approach for many non-polio-endemic LMICs in sub-Saharan Africa.

Our results are generalisable to different geographies. Using the modelled population size as a guide alongside national RI coverage and historical pSIA schedules, many geographies can be mapped to table 5. For countries with population sizes smaller or larger than our modelled population, model estimates can be scaled up or down.

Further, below 66.6% RI, both pSIA strategies are cost-effective and avert a substantial number of DALYs, outweighing the increased number of expected VAPP cases. Countries such as Madagascar or Angola, where 55% and 42% of the population under 5 years, respectively, are vaccinated with three or more DTP doses, have many subpopulations that could benefit from regular pSIAs. In Ghana and Sierra Leone, for example, future SIAs would not seem necessary as RI coverage of three doses of bOPV and one dose IPV exceeds 90% without reliance on historic pSIAs, unless there are subpopulations with substantially lower coverage. The proportion of the population in Malawi that has received three doses of bOPV peaked in 2011 but has been unstable since, falling to 83% in 2016,^19^ with no historic reliance on pSIAs.

Our study has implications for global polio eradication decision-making and health policy. Decisions on vaccination strategies should consider the combined perspective of the health system and GPEI rather than relying solely on one perspective. From the health system perspective, pSIAs are no longer cost-effective when RI coverage exceeds 66.6%. This is an important finding because it captures a country’s health system perspective, which often has competing health priorities. Despite the high costs and increased VAPP, reduced outbreak probability under annual pSIAs is an important consideration for polio eradication but should be considered alongside country health system perspectives to capture the full complexity of benefits and trade-offs (outbreak risk and cost-effectiveness) of costly pSIAs. Should the GPEI adopt an annual pSIA strategy irrespective of estimated RI and importations? Annual pSIAs that include all children under 5 in LMICs in sub-Saharan Africa would consume most of the GPEI annual budget for activities and would be an inefficient use of funds, potentially reducing funds for other activities (surveillance, vaccination against other serotypes). However, prioritising pSIAs in countries with low RI and perceived risk of introductions is a necessary compromise to which GPEI already adheres, and here we provide a framework to support decision-making. Renewed commitment by donors was requested in 2022^34^ considering the 2022–2026 Strategic Plan, and these commitments remain essential to resource the activities needed to meet the objectives of polio eradication, including interrupting WPV transmission.

Our study has limitations. The main results assume an R_0_ of 3 in a homogenous population (both with respect to transmission and population immunity), two imported infections per year and SIAs reaching 25% of children missed by RI. If SIAs reach up to 50% of zero-dose children, the impact of SIAs on reducing outbreak risk is further increased. Consequently, for the same costs, a better outcome is achieved (online supplemental appendix p20). Assuming a higher R_0_ and increasing the frequency of importations would also increase the outbreak risk (online supplemental appendix pp 21–22). One of the most uncertain inputs of the analysis is the importation rate: as poliovirus infection is typically asymptomatic, this is not directly observable, and due to the changing epidemiology of polio globally, the importation rate will vary over time. We have not considered population heterogeneity. If there are pockets of the population with higher rates of transmission and/or lower vaccination coverage, then the probability of an outbreak occurring would increase. We only model children under 5, given the limited but uncertain extent to which older children and adults contribute to WPV transmission, which may underestimate total expected cases. Research suggests no evidence of imperfect intestinal immunity in adults and older children in the transmission of WPV across different locations, which supports our modelled target population,^16^ but in the future, more research is needed to better understand context-specific transmission by older ages.

While we have used cost-effectiveness thresholds based on the growth in life expectancy and health expenditures,^26^ alternative thresholds based on health opportunity costs could be further explored.^35^,^37^ We do not consider the costs of further delaying the eradication timeline through outbreaks, or the societal implications of outbreaks on polio eradication, both of which may further emphasise the need to implement pSIAs even when the outbreak risks are small. We also do not include the impact of joint SIAs that might deliver other interventions or vaccines alongside OPV, as these joint campaigns occur less frequently and are programmed differently than polio-specific SIAs. By limiting our analysis to a 5-year time horizon, we underestimate the benefits of SIAs (particularly pSIAs) as they will increase the likelihood of eradication, meaning that control efforts after eradication can be scaled back. However, this time horizon was chosen to specifically align with the current GPEI strategic plan for imminent programmatic decisions. Further, the pSIA health system costs only consider the geographical remit stated in the model and ignore the potential for further international spread. International spread would be far more likely with larger outbreaks; consequently, the health system costs are underestimated.

Our findings suggest that SIAs may become less cost-effective in settings where RI coverage is higher (>65%) because the incremental benefits of mass campaigns diminish when population immunity is already high. This raises questions about whether the funds allocated to SIAs might be better used to strengthen health systems, expand RI coverage further, or address other pressing health challenges, which often take a significant toll in sub-Saharan Africa. Requiring LMICs to continue polio SIAs could be seen as an imposition by global health authorities, particularly if these countries are burdened with funding or logistical responsibilities they cannot afford. Ethical principles of equity and reciprocity suggest that wealthier countries or global health initiatives should bear a significant share of the financial and operational burden for SIAs in resource-constrained settings. From a global perspective, achieving polio eradication would be a public good, benefiting all nations by eliminating the disease permanently. However, in LMICs where health resources are limited, prioritising eradication efforts over other critical health needs may not align with local public health priorities. On the other hand, reducing or eliminating SIAs prematurely in countries with suboptimal RI coverage may increase the risk of polio outbreaks. This could result in higher costs and disease burden in the future, potentially undermining broader public health efforts and delaying eradication—a global objective.

The synergy between what is cost-effective and what is necessary for an eradication programme is complex. For example, our findings that SIAs are not cost-effective above 65% RI coverage make sense when considering risk in polio-free settings with competing health priorities. However, from an eradication perspective, other cost considerations become relevant. Reducing the frequency of pSIAs in a geography with 80% RI coverage makes sense from a cost-effectiveness perspective, but at this level of RI coverage, outbreak risk persists if the importation of WPV1 were to occur. From the global perspective, investing in pSIAs results in a greater probability of polio elimination, but still requires justification in a pragmatic environment of finite resources. These motivations align with the game theoretic approach proposed by Barret *et al* such that global eradication only succeeds if the country with the weakest elimination programme is successful and that success depends on mutual assurance.^38^ Many non-endemic countries in sub-Saharan Africa have an incentive to maintain the elimination of polio, but domestic funding is limited and GPEI is left to support the budget gaps in polio programming.^38^ Well-resourced countries that have eliminated polio have an incentive to financially support or incentivise less-resourced endemic countries to eliminate polio to realise the full potential of their investments already made, and therefore financially support GPEI.

In conclusion, we assessed the outbreak risk and cost-effectiveness of different vaccination strategies and critically assessed the risks associated with adopting different strategies, given baseline RI coverage. Decisions made solely based on fixed budget, cost-effectiveness or burden reduction may not fully capture all consequences or benefits associated with adopting a particular vaccination strategy. Urgently, as importations of WPV1 remain a threat to the African region, this analysis serves as a valuable tool to estimate risk and plan vaccination activities across a range of settings at risk of importation of WPV1 cases.

## supplementary material

10.1136/bmjgh-2024-016013 online supplemental appendix 1

## Data Availability

Data are available in a public, open access repository.
