# Supplementary material for: Vaccination strategies against wild poliomyelitis in polio-free settings: outbreak risk modelling study and cost-effectiveness analysis
Source: BMJ Glob Health. 2025 Mar 22;10(3):e016013. doi: 10.1136/bmjgh-2024-016013 (PMC11931904; doi:10.1136/bmjgh-2024-016013)
Supplement: online supplemental appendix 1 [file bmjgh-10-3-s001.pdf]

Appendix

Section 1: Model structure, parameters and limitations

Execution of pSIAs and oSIAs

SIAs, including both pSIAs and oSIAs include door-to-door delivery of OPV to children under five years of age. Before an SIA occurs, a target population for the activity is set and vaccinators operate within this target geography for the activity. OPV does not require a cold chain and therefore allows for vaccinators to vaccinate hard-to-reach children who would otherwise be missed by routine immunisations. Once a child has been vaccinated, the dose is recorded on a health card or the child's finger is marked with ink to indicate to the vaccinators that the child has already received a dose during the SIA. This prevents vaccinators from vaccinating the same children twice. This process is used in both pSIAs and oSIAs. Therefore, the transition of a child between model compartments following a vaccine dose is identical for pSIAs and oSIAs. The parameters such as SIA coverage, vaccine efficacy and seroconversion are the same for pSIAs and oSIAs

The difference between pSIAs and oSIAs is how they are funded and planned. oSIAs are emergency responses to outbreaks and therefore, occur rapidly following an outbreak (at least within 90 days) only in areas affected by the outbreak. Sometimes, a buffer area, or a geography surrounding the outbreak, can also be vaccinated during an oSIA. pSIAs occur as National Immunisation Days (NIDs) or Subnational Immunisation Days (SNIDs) and usually cover a larger geographical area than oSIAs. These funding and planning differences are important for the economic aspects of this analysis, but do not alter any model transitions in the SIR model below. For calculation of historical SIAs, we assumed that pSIAs included NIDs or SNIDs with bOPV (or trivalent OPV (tOPV) pre-2016) that did not occur within 365 days of a WPV1 or VDPV1 detection by AFP surveillance or ES. Any SIA that occurred within this interval was not included in the overall count of pSIAs.

Geographical setting modelled

In this analysis, we model a hypothetical geography for a non-polio endemic LMIC in sub-Saharan Africa with a population size of approximately 8 million children under five years of age. There are a few reasons we did not model a specific country. Firstly, WPV1 importation into Africa is not, in 2024, a common occurrence. Therefore, we cannot fit this model to actual WPV1 importation data in many geographies. We saw an unexpected, albeit understandable, importation event in 2021, which highlighted the need for other non-endemic countries in AFRO to evaluate their current vaccination strategies given baseline RI coverage and importation risk, given onward transmission from the Malawi importation event. One benefit of using a hypothetical setting is that the implications of this model can be applied to a range of countries even in the absence of an importation to predict risk and understand the benefits and risks of adopting different SIA strategies.

Model structure

A stochastic non-linear mathematical model was used to simulate polio transmission dynamics, whereby infectious individuals develop either asymptomatic (I, or infectious, compartment), or symptomatic infection (C, or case, compartment), both of which are assumed to be infectious. Both infections and cases recover to the Rn compartment. In the model, children are either susceptible (S0 compartment), fully vaccinated and protected from poliovirus infection (Rv compartment) or have received an incomplete vaccination series (less than 3 bOPV doses + 1 IPV dose, modelled separately). Children who were not vaccinated via RI, can receive additional doses of bOPV vaccine through either pSIAs or oSIAs. Each subsequent dose of vaccine corresponds additional protection and an opportunity

for a child to seroconvert and be considered fully protected from poliovirus infection (corresponding to each of the Sn tiers).

The left side of Appendix Figure 1 corresponds to children missed by RI who have an opportunity for additional bOPV doses via SIAs. Progression through the model to different Sn compartments happens only at the time of an SIA.

The right side of Appendix Figure 1 corresponds to RI and happens daily, when life births enter the population. Vaccination via RI is assumed to occur upon entering the model. Children vaccinated via RI are assumed to receive a sequential schedule of both bOPV and IPV, whereby they are assumed to seroconvert and be protected from poliovirus (transitioning to Rv compartment). Only children previously vaccinated via RI are eligible for vaccination with IPV and IPV is provided alongside the third dose of bOPV via RI in most African countries.

To guide understanding of the model diagram, the compartments correspond to the following:

- S0 = susceptible to poliovirus, no vaccine received
- I = infectious, but asymptomatic
- C = infectious and polio (AFP) case
- Rn = recovery via natural infection
- S_n = susceptible to poliovirus infection, but have received *n* bOPV doses via SIAs (S_{3sia} explicitly refers to children who received the 3rd bOPV dose via SIAs not RI)
- $RI_{3bOPV + IPV}$ = vaccinated with 3 bOPV doses + IPV at birth via RI
- $S3_{ri}$ = vaccinated with bOPV, but missed IPV dose

Event table for the SIR model

Appendix table 1 shows transitions and transition probabilities in the stochastic SIR model. The compartments and probabilities correspond to Appendix figure 1 and Appendix Table 3.

Appendix Table 1. Model transitions and transition probabilities in the stochastic SIR model

S0 → $((1 – \rho) * \phi) * (\beta * S0 * (I + C)) / (S0 + S_n + RI_{3bOPV + IPV} + Rv + I + C + Rn)$ → I
S0 → $\rho * \phi * (\beta * S0 * (I + C)) / (S0 + S_n + RI_{3bOPV + IPV} + Rv + I + C + Rn)$ → C
S_n → $((1 – \rho) * \phi) * (\beta * S_1 * (I + C)) / (S0 + S_n + RI_{3bOPV + IPV} + Rv + I + C + Rn)$ → I
S_n → $\rho * \phi * (\beta * S_1 * (I + C)) / (S0 + S_n + RI_{3bOPV + IPV} + Rv + I + C + Rn)$ → C
$RI_{3bOPV + IPV}$ → $((1 – \rho) * \phi) * (\beta * RI_{3bOPV + IPV} * (I + C)) / (S0 + S_n + RI_{3bOPV + IPV} + Rv + I + C + Rn)$ → I
$RI_{3bOPV + IPV}$ → $\rho * \phi * (\beta * RI_{3bOPV + IPV} * (I + C)) / (S0 + S_n + RI_{3bOPV + IPV} + Rv + I + C + Rn)$ → C
I → $\gamma * I$ → Rn
C → $\gamma * C$ → Rn

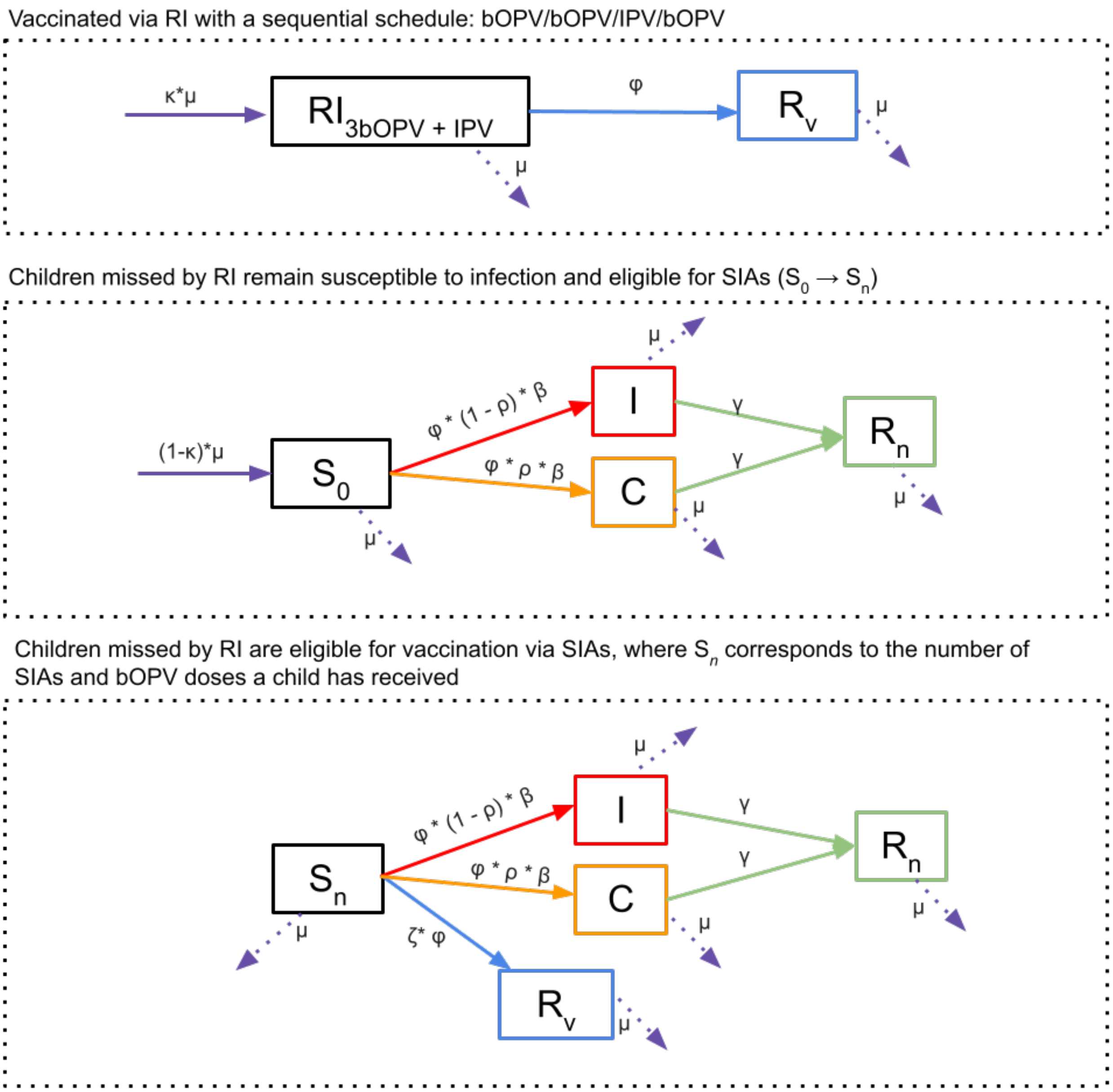

Appendix Figure 1. Model structure of the extended SIR model. Births enter the population either via the S0 compartment or the $S3_{ri}$ compartment, assuming vaccination with three bOPV doses occurs via RI at the time of birth. Children can exit the population from any compartment via death. Children missed by RI at birth remain in the S0 compartment, with potential for vaccination with one dose of bOPV via pSIA or oSIA. Each of the SIA tiers (S_n), where n corresponds to the number of bOPV doses received via an SIA, allow for potential seroconversion and transition to the Rv compartment according to assumed bOPV vaccine efficacy. Children who do not seroconvert following bOPV during an SIA progress to the next vaccination tier, n+1, and await subsequent SIA vaccination. Each of the SIA tiers remain susceptible to infection. In this model we assume that bOPV coverage = IPV coverage.

Model compartments and transitions

A review of the immunogenicity of vaccination schedules with both IPV and three doses of bOPV indicate high rates of seroconversion alongside some geographical variability [1] and sustained protection against paralytic poliomyelitis [2]. Accordingly, we assume seroconversion and transition to the Rv compartment is 100% for children who received three doses of bOPV and one dose of IPV and no waning of protection. The assumptions described here about vaccination activities provide a 'steady state' immunity profile which we use to explore the effects of polio introductions. We assume that once five years of age is reached, children are no longer relevant to poliovirus transmission, due to the high likelihood of immunity against infection due to vaccination.

Appendix Table 2. Model compartments and corresponding explanation of compartmental transitions and assumptions

Compartment	Assumption
S_0	Susceptible individuals in this compartment have not been vaccinated, i.e., zero doses received of both bOPV and IPV. The proportion of unvaccinated individuals born into this compartment depends on assumed RI coverage. From S_0, individuals exposed to 1 dose of bOPV via an SIA progress to S_n. Individuals who stay in S_0 remain susceptible to infection.
S_n	Individuals in this compartment have received *n* doses of bOPV via an SIA. From each S_n compartment, a certain proportion will seroconvert after vaccination and transition to Rv, according to bOPV vaccine efficacy (see parameter ϕ below). While those that did not seroconvert stay in S_n and remain susceptible to infection (adjusted by a factor of ϕ for protection received by *n* doses) and are eligible for future vaccination in a subsequent SIA. After 4 bOPV doses, all children are assumed to seroconvert and transition to Rv after completing a full series of vaccinations.
$RI_{3bOPV + IPV}$	Assuming bOPV coverage = IPV coverage. Children in this compartment are vaccinated via RI with a sequential schedule of OPV/OPV/IPV/OPV. It is assumed after 3 doses of bOPV and 1 dose IPV 100% of children seroconvert and transition to the Rv compartment and are also protected from paralysis.
$S3_{ri}$	Individuals in this compartment received 3 doses of bOPV at birth via RI. From $S3_{ri}$, a certain proportion will seroconvert after 3 doses and transition to Rv, according to bOPV vaccine efficacy (see parameter ϕ below in Appendix Table 3). Of those vaccinated with IPV, 100% are assumed to seroconvert and transition to Rv.
I	Individuals in this compartment are infectious, but asymptomatic. They can spread infection to others but are not a paralytic case of polio. Importations of infection who enter the population enter via this compartment.
C	Individuals in this compartment are a paralytic case of polio. They can spread infection to others and trigger an outbreak response campaign in the baseline strategy.
Rn	Individuals in this compartment have recovered from natural infection and are assumed immune to subsequent infection.
Rv	Individuals in this compartment have received sufficient vaccination to result in seroconversion and are assumed immune to subsequent infection.

Model parameters, assumptions and transition probabilities

Appendix Table 3. Table of model parameters and corresponding values and assumptions

Parameter	Value	Assumption
γ	1/8	γ = recovery rate – the infectious period for polio is between 7-10 days before AND after symptoms. Here, we took the average, 8 days that an individual can remain in the I or C compartment, before transitioning to R_n
R_o	3	Basic reproductive number. For polio, the basic reproductive number, $R_{0,}$ can vary substantially between locations depending on sanitation and hygiene conditions [3].
β	0.375	The effective contact rate, which affects the transition from the susceptible compartment to the infected compartment, and the rate of recovery, which affects the transition from the infected compartment to the recovered compartment.<br>If, $R_o = \beta / \gamma$ Then, if R_o is 3, and γ is 1/8, then β is 0.375
ρ	1/200	Case to infection ratio for WPV. The WPV1 case to infection ratio was assumed to be 1:200, which is consistent with estimates for poliovirus serotype 1 [4].
κ	Varied between 0.25 – 1.0	Proportion that received 3 doses of bOPV via RI
ζ	0.25	Proportion of the target population reached by the SIA, those missed by the campaign are represented by $1-\zeta$
ω	Varied between 0.25 – 1.0	Proportion exposed to IPV vaccination
ϕ	$1 - ((1 - 0.5)^{doses})$	Vaccine effectiveness of bOPV for protection against serotype 1, i.e., the proportion of the population that seroconvert and transition to Rv assuming vaccine efficacy (VE) is 50%
μ	$5{*}10^{-4}$ * population	Birth rate = death rate<br>4,000 live births per day in a country of 8 million is the average number of live births across African countries, assumptions on the equal birth and death rate are in line with other research using a similar hypothetical population [5]
Herd immunity	$1\text{-}1/R_0$	Using an R_0=3, the herd immunity threshold would be assumed to be reached at 66.67% RI coverage

Model framework

It is important to discuss the model framework used in this analysis and associated pros and cons of the simplistic model structure and assumptions. Model simplicity allows for easy interpretation and is applicable to a range of settings. Here, we model a hypothetical population size for a LMIC in sub-Saharan Africa using certain fixed parameters, but the simple model structure can be easily adapted to fit specific countries, or even subnational populations. The model compartments and transitions are easy to understand by a wide range of audiences, not just mathematical modellers and the SimInf package allows for easy adaptation of scheduled vaccination activities to local contexts. SimInf uses continuous-time Markov chains using the Gillespie stochastic simulation algorithm (SSA) to integrate infection dynamics and incorporates available data such as births, deaths or vaccination as scheduled events.

The simplicity of the model does give rise to several limitations. In this model, we assume homogenous mixing, assume that the same polio programme has been in place for 50 years prior to model initiation, assume SIAs reach 25% of children unvaccinated by RI, assume a single value for R_0 and other model parameters with no uncertainty. Of particularly importance is heterogeneity in the population structure. If there are pockets with higher rates of transmission or lower vaccination coverage (or both) then this would increase the likelihood of outbreaks and thus decrease the likelihood that eradication will be achieved. Sensitivity analyses in Appendix Section 4 further explore varying assumptions about SIA target population, importation rate and R_0. In summary, under different assumptions, model outputs across vaccination strategies are only affected when RI coverage is very low and therefore, support the assumptions used in the main analysis.

Further, the model structure assumes that children are vaccinated when they are born into the population, not accounting for infection prior to vaccination. Because the average age of infection with poliomyelitis occurs after the vaccination schedule of bOPV given at 6, 10 and 14 weeks of age alongside the effect of maternal antibodies, this limitation is unlikely to impact the expected number of AFP cases. However, because the size of the birth cohort vaccinated may be overestimated in the absence of infant mortality assumptions, the costs of RI may be over-estimated here. Additionally, we do not include indirect costs of vaccination, such as opportunity costs of time spent for vaccination, which may result in an underestimation of costs across all strategies. The indirect benefits of these additional interventions (such as productivity increases from averting polio cases) are also not quantified in the costs and benefits of SIAs described here, therefore the deaths and burden averted are polio specific and exclude indirect economic effects, in contrast to some other analyses.

Section 2: Model and cost assumptions

IPV and OPV coverage estimates

This WEUNIC data shows the relationship between IPV and OPV3 coverage (three doses of bOPV vaccine) for twenty-five countries in sub-Saharan Africa. IPV was introduced later in time in all countries, and although at the time of introduction IPV coverage was lower than OPV3 coverage in most countries, in 2021, IPV and OPV3 coverage was roughly equal across all countries. This data supports the model assumption that IPV coverage = OPV3 coverage, or all children that receive a third dose of bOPV, also received IPV via RI.

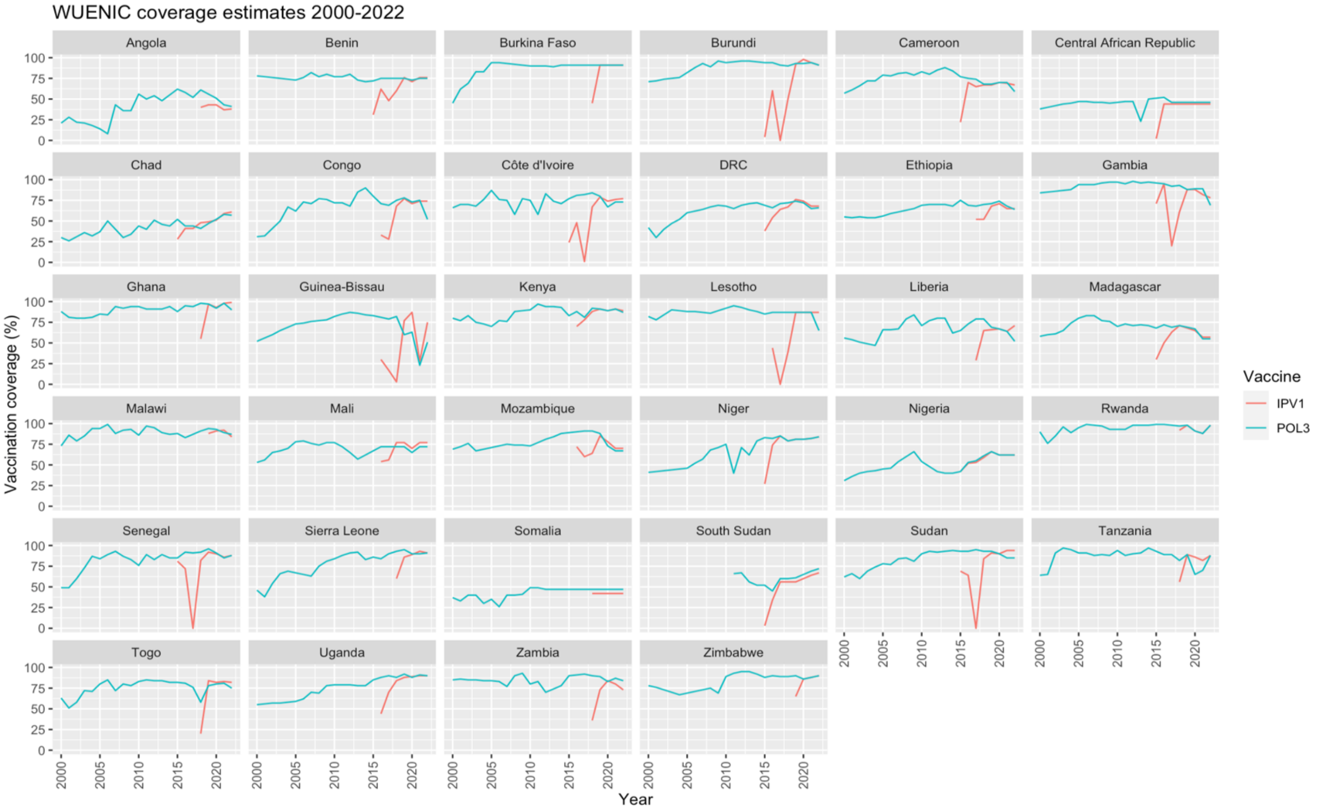

Appendix Figure 2. WUENIC coverage estimates 2000-2021 for OPV3 and IPV1 across Sub-Saharan African countries. The strong correlation between OPV3 and IPV1 coverage estimates in 2020 and 2021 (when scale-up of IPV implementation was reached) support model assumptions that OPV coverage is equal to IPV coverage. DRC = Democratic Republic of Congo.

Sample size calculation

The United Nations World Population Prospects (WPP) data was used in our sample size calculation [6]. We used WPP data from 2021 to calculate the mean under-5 population size in 25 Sub-Saharan countries: Burundi, Guinea, Benin, Zimbabwe, Senegal, Malawi, Zambia, Somalia, Chad, Burkina Faso, Mali, Madagascar, Côte d'Ivoire, Cameroon, Ghana, Niger, Mozambique, South Africa, Angola, Kenya, Sudan, Uganda, United Republic of Tanzania, Democratic Republic of the Congo, Ethiopia, Nigeria. The mean under-5 population size was 8,000,000 and the range of the data was (2,500,000 – 41,000,000). Given this large range of population sizes across countries, we chose to use the mean under-5 sample size amongst these countries as our target-population size for this modelling. Historical pSIAs and RI coverage of an additional 10 countries with smaller under-5 populations are referenced in the manuscript to give regional context and our results are still relevant for these geographies, but absolute cases and costs need to be scaled according to population size.

Cost assumptions

We assume costs of SIAs are the same across the entire modelled time horizon. Costs associated with vaccine doses and the number of children vaccinated during an SIA are estimated using the entire target population of eight million children under five years of age, even though in practice, the true proportion of children reached during SIAs is often much lower due to operational challenges and other logistical shortcomings [7]. As model outputs captured the variability across all stochastic simulations, costs for each individual simulation were calculated across all strategies. The average expected costs for each strategy were then obtained by taking the mean cost across all simulations for each corresponding strategy. The GPEI costs include those associated with SIAs and IPV in RI. Treatment costs associated with paralytic polio (including VAPP) are paid for by the country and included as health care system costs; specific equations used in the costing estimates can be found below in Appendix Table 4.

Literature has shown that costs associated with RI administration can vary across RI coverage for different antigens [8, 9], but usually the threshold cut-off is below and above 80% RI coverage and explicit evidence of this differential for polio is not well documented. Accordingly, we use one value for costs associated with RI administration for polio as documented by Kalkowska et al.

Appendix Table 4. Table of cost inputs and corresponding values and assumptions

Item	Value (USD$2023)	Assumption / reference
bOPV dose	$0.18	Average cost for a GAVI country [9], price per dose in a 10-dose vile
IPV dose	$2.00	For a GAVI country 10-dose vile and translated into USD$2023 [10]
RI OPV admin	$1.06*3 doses	Costs associated with administration, procurement and storage of OPV for use in RI. 2019 costs were obtained from appendix Table A1 [11]. We assume costs and wastage are based on the size of the entire birth cohort.
RI IPV admin	$2.00	Costs associated with administration, procurement and storage of IPV for use in RI. 2019 costs were obtained from appendix Table A1 [11]. We assume costs and wastage are based on the size of the entire birth cohort.
pSIA	$0.50/child	Includes operational costs, social mobilisation and administration. We assume that SIAs are planned to reach 100% of the population and costed accordingly but the actual population vaccinated is 25% due to operational challenges. Costs are obtained from GPEI
oSIA	$1.0/child<br><br>Range: $0.50 – $1.50/child	Includes costs associated with emergency response to an outbreak. We assume that SIAs are planned to reach 100% of the population and costed accordingly but the actual population vaccinated is 25% due to operational challenges. Costs are estimated based on GPEI costs for pSIAs
VAPP rate	Dependent on dose and vaccination schedule	1st dose: 0.9 VAPP cases/1 million bOPV doses administered, a 6.6-fold greater risk than following subsequent doses [12]<br><br>The risk of VAPP following OPV vaccination is reduced if IPV has been received. We assume no risk of VAPP associated with IPV vaccination and a 53% reduction in VAPP cases following bOPV if IPV has already been received

DALY	14/paralytic case	Assume AFP case = VAPP case
Paralytic case	$700/case	Assume AFP case = VAPP case, cost is for a low-income country [8]
bOPV wastage RI	Range 10-20%	Range of estimated wastage of bOPV in RI [13, 14]
bOPV wastage SIA	Range 5-15%	Range of estimated wastage of bOPV in both pSIAs and oSIAs [13, 14]
IPV wastage RI	Range 5-20%	Range of estimated wastage of IPV in routine settings. Range varies based on vial size and setting [13, 14]
Health care system & non-GPEI costs		Treatment costs (red) + RI OPV costs (blue)<br><br>*(Cost per AFP case * AFP cases) + (Cost per VAPP case * VAPP cases)*<br>+<br>*(RI coverage *(New-borns eligible for bOPV vaccination * total doses received per child) * (Cost per dose of bOPV + RI delivery cost per dose of bOPV) * (1 + bOPV wastage rate for RI))*
GPEI costs		pSIA costs (green) + oSIA costs (purple) + RI IPV costs (orange)<br><br>*(SIA coverage * (Target population† * Number of pSIAs) * (Cost per dose of bOPV + pSIA delivery cost per dose of bOPV) * (1 + bOPV wastage rate for SIAs))*<br>+<br>*(SIA coverage * (Target population† * Number of oSIAs) * (Cost per dose of bOPV + oSIA delivery cost per dose of bOPV) * (1 + bOPV wastage rate for SIAs))*<br>+<br>*(RI coverage * (New-borns eligible for vaccination) * (Cost per dose of IPV + RI delivery cost per dose of IPV) * (1 + IPV wastage rate for RI))*<br><br>† Target population for pSIAs and oSIAs refers to all children under five years of age

Calculation of Incremental Cost-Effectiveness Ratios (ICERs)

ICERs were calculated using the following equation:

$$ICER = \frac{(costs\ of\ pSIA\ strategy - costs\ of\ baseline\ strategy)}{(DALYs\ averted\ by\ pSIA\ strategy)}$$

Mean ICERs were calculated using the mean costs and DALYS of each vaccination strategy for each RI coverage level across all model simulations. Then to get uncertainty, we calculated an ICER for each of the simulations. We extracted the lowest (Democratic Republic of the Congo), median (Benin) and highest (South Africa) cost-effectiveness thresholds among the 25 low, lower middle and upper middle-income sub-Saharan African countries used in our sample size calculation from supplementary table 3 by Pichon-Riviere A et al. 2023 [15]. The results are provided not to drive a specific investment decision within a health technology assessment (HTA) or other decision-making framework (as a cost-effectiveness analysis for a new drug or vaccine would be), but to provide general evidence to inform policy making. GDP data was obtained from the World Bank database [16].

Section 3: Stochastic simulations result in variable outcomes across strategies

Variable number of outbreaks across all strategies

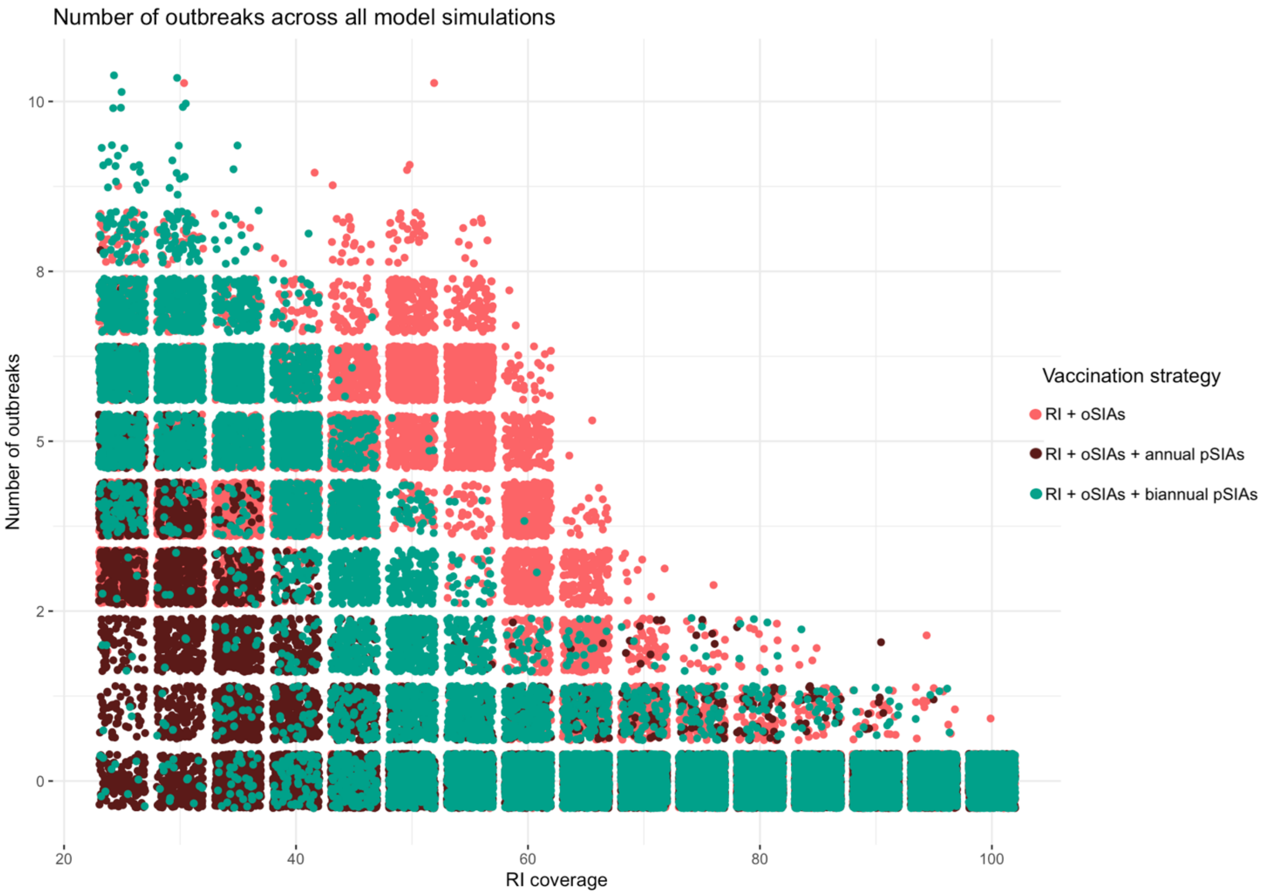

Appendix Figure 3. Number of outbreaks in each individual model simulation for each vaccination strategy, among simulations with ≥1 paralytic polio case. The low number of outbreaks at RI coverage levels >70% correspond to a lower probability of an outbreak, which was lowest in the annual pSIA strategy.

The stochasticity of epidemics means that there is considerable variability in outcome for identical assumptions focussing on simulations that had ≥1 paralytic polio case (Appendix Figure). We define an outbreak as at least one case of paralytic poliomyelitis, which we assume would present as acute flaccid paralysis (AFP). To count the total number of outbreaks, all cases that occurred within 90 days of the first case were considered part of the same outbreak. Cases that occurred in subsequent intervals of 90 days were considered part of different independent outbreaks. Even though some cases that are allocated to different outbreaks may in fact be linked or a part of the same outbreak, the 90-day threshold aligned with the schedule of oSIAs and was used as a consistent unit to count outbreaks across strategies. This method of counting outbreaks may incorrectly attribute linked cases from the same outbreak as independent outbreaks but was taken to reduce model complexity. As standard operating procedures for polio outbreaks recommend an oSIA in affected areas within 90-days of the first case, if an outbreak is not stopped or subsequent cases arise later, this would trigger a subsequent oSIA, a chronology which is accurately accounted for in this analysis. In the absence of having uniquely identifiable outbreaks within the model, simplifying the outbreak count by using a 90-day cut-off is a limitation that may over-estimate the total number of outbreaks, however, the counting methodology is consistent across all evaluated strategies.

Trends in VAPP and DALYs

Vaccine associated paralytic polio (VAPP) occurs when a strain of poliovirus that has genetically mutated from the original attenuated vaccine strain contained in OPV causes paralysis [17]. VAPP can occur following vaccination with OPV, or it can also occur in a close unvaccinated or non-immune contact of a vaccine recipient who excretes the mutated virus [17]. VAPP rate across settings with and without SIAs has been shown to vary [18], although this variation is not well understood or documented. OPV administration following IPV receipt is associated with a further risk reduction of VAPP [12] and is accounted for in model assumptions (Appendix Table 4). Expected VAPP cases over the same interval are greatest in the annual pSIA strategy, corresponding to the strategy with the greatest number of vaccine doses administered (Appendix Figure 4). The annual pSIA strategy administers the greatest number of vaccine doses and is the strategy that results in the fewest expected WPV cases over five-years (main text figure 2A). At RI coverage levels below 70%, DALYs incurred are greatest in the baseline strategy, the strategy with the greatest number of expected AFP cases. However, at RI coverage levels >70%, the average number of VAPP cases exceeds the number of WPV1 cases in all pSIA strategies, which drives these two strategies to have a greater number of estimated DALYs lost than the RI strategy alone. Expected DALYs in the annual pSIA strategy are greatest when RI coverage exceeds 70% because the number of pSIAs remains constant over time, resulting in more VAPP cases and consequently, a greater number of DALYs incurred. In contrast, above 70% RI coverage, the baseline strategy has fewer WPV1 cases and no or fewer oSIAs, resulting in fewer VAPP cases and therefore fewer expected DALYs incurred.

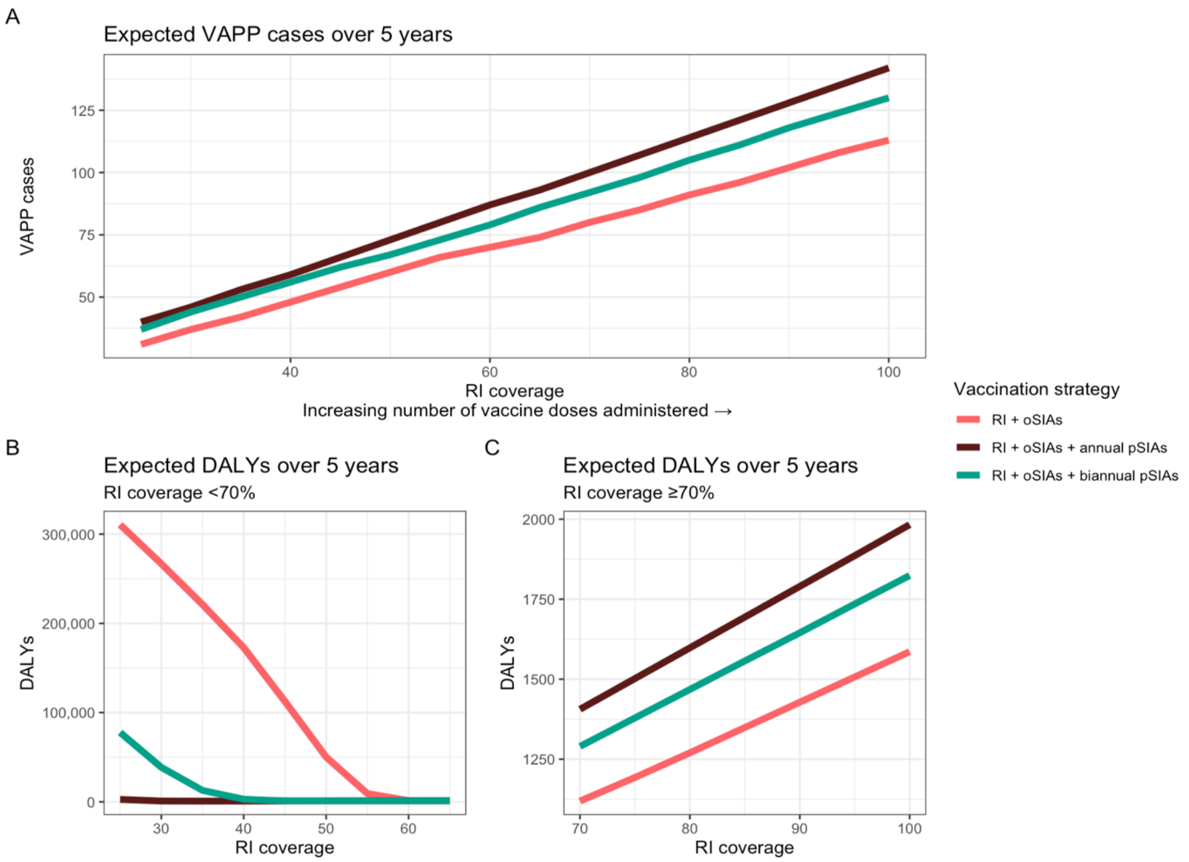

Appendix Figure 4. Average number of expected VAPP cases and DALYs per capita over 5 years amongst model simulations that reported at least one outbreak. The increase in VAPP cases is related to the increase in vaccine doses administered.

Appendix Table 5. Health system perspective: table of cases, VAPP, DALYS and costs across all strategies. Uncertainty in cases and associated costs is presented as 95% credible intervals (CI). Here, the mean values are obtained from taking the mean across all model simulations, not just amongst the simulations with an outbreak (as was done in table 2 in the main text).

RI (%)	Strategy	Mean cases	Cases lwr 95% CI	Cases upr 95% CI	Mean outbreaks (range)	Mean VAPP	Mean DALYS	Mean total costs ($)	Total costs ($) lwr 95% CI	Total costs ($) upr 95% CI
25	Annual pSIA	156	129	183	4 (0—8)	40	2742	8065554	7955389	9028399
30	Annual pSIA	20	16	24	2 (0—6)	46	930	9560618	9545889	9652046
35	Annual pSIA	3	3	4	1 (0—5)	53	784	11139031	11136390	11150405
40	Annual pSIA	1	1	1	1 (0—3)	59	843	12727687	12726890	12730621
45	Annual pSIA	0	0	0	0 (0—3)	66	931	14317772	14317391	14319951
50	Annual pSIA	0	0	0	0 (0—3)	73	1024	15908119	15907891	15909613
55	Annual pSIA	0	0	0	0 (0—2)	80	1118	17498506	17498392	17499412
60	Annual pSIA	0	0	0	0 (0—2)	87	1214	19088967	19088893	19089831
65	Annual pSIA	0	0	0	0 (0—2)	93	1310	20679448	20679394	20680270
70	Annual pSIA	0	0	0	0 (0—2)	100	1406	22269934	22269894	22270717
75	Annual pSIA	0	0	0	0 (0—2)	107	1502	23860434	23860395	23861221
80	Annual pSIA	0	0	0	0 (0—2)	114	1598	25450918	25450896	25451599
85	Annual pSIA	0	0	0	0 (0—1)	121	1694	27041413	27041397	27041399
90	Annual pSIA	0	0	0	0 (0—1)	128	1790	28631903	28631897	28631899
95	Annual pSIA	0	0	0	0 (0—1)	135	1886	30222399	30222398	30222399
100	Annual pSIA	0	0	0	0 (0—0)	142	1983	31812928	31812928	31812928
25	Biennial pSIA	5520	5351	5689	6 (0—10)	37	77800	11818435	8287477	15535884
30	Biennial pSIA	2718	2577	2859	6 (0—10)	44	38663	11447281	9561613	14853006
35	Biennial pSIA	866	797	935	5 (0—9)	50	12822	11740950	11133068	13865749
40	Biennial pSIA	154	137	171	4 (0—8)	56	2936	12832320	12723213	13448221
45	Biennial pSIA	16	15	18	3 (0—7)	62	1093	14325840	14313359	14387991
50	Biennial pSIA	3	2	3	1 (0—5)	67	981	15905929	15903505	15915663
55	Biennial pSIA	1	1	1	1 (0—4)	73	1035	17494354	17493651	17497300
60	Biennial pSIA	0	0	0	0 (0—4)	79	1116	19084100	19083798	19085739
65	Biennial pSIA	0	0	0	0 (0—2)	86	1203	20674109	20673944	20674993
70	Biennial pSIA	0	0	0	0 (0—2)	92	1290	22264176	22264091	22265056
75	Biennial pSIA	0	0	0	0 (0—2)	98	1379	23854299	23854237	23855158
80	Biennial pSIA	0	0	0	0 (0—2)	105	1468	25444422	25444384	25445241
85	Biennial pSIA	0	0	0	0 (0—2)	111	1557	27034555	27034531	27035333
90	Biennial pSIA	0	0	0	0 (0—1)	118	1645	28624688	28624677	28624679
95	Biennial pSIA	0	0	0	0 (0—1)	124	1735	30214829	30214824	30214825
100	Biennial pSIA	0	0	0	0 (0—0)	130	1824	31804991	31804991	31804991
25	Baseline	22158	22071	22246	4 (2—9)	31	310649	23460911	21665597	25612092
30	Baseline	19021	18938	19105	4 (3—10)	37	266811	22854690	20515728	25133539
35	Baseline	15753	15664	15842	4 (3—8)	42	221134	22156506	19692406	24125425
40	Baseline	12296	12182	12409	5 (0—9)	48	172812	21326123	18539293	23184345
45	Baseline	7990	7859	8120	5 (0—9)	54	112616	19901986	16503382	22298018
50	Baseline	3524	3399	3649	6 (0—10)	60	50181	18365947	15986096	21224605
55	Baseline	598	546	650	5 (0—8)	66	9290	17907087	17486193	19487919
60	Baseline	30	26	34	3 (0—7)	70	1399	19098238	19075847	19199688
65	Baseline	2	2	2	1 (0—5)	74	1073	20667602	20665501	20674099
70	Baseline	0	0	0	0 (0—3)	80	1119	22255585	22255155	22257731
75	Baseline	0	0	0	0 (0—3)	85	1193	23844979	23844808	23845898
80	Baseline	0	0	0	0 (0—2)	91	1270	25434546	25434462	25435474
85	Baseline	0	0	0	0 (0—2)	96	1349	27024160	27024116	27025050
90	Baseline	0	0	0	0 (0—1)	102	1428	28613790	28613770	28613770
95	Baseline	0	0	0	0 (0—2)	108	1507	30203435	30203424	30203424
100	Baseline	0	0	0	0 (0—1)	113	1586	31793079	31793078	31793078

Appendix Table 6. GPEI perspective: table of cases, VAPP, DALYS and costs across all strategies. Uncertainty in cases and associated costs is presented as 95% credible intervals (CI). Here, the mean values are obtained from taking the mean across all model simulations, not just amongst the simulations with an outbreak (as was done in table 2 in the main text).

RI (%)	Strategy	Mean cases	Cases lwr 95% CI	Cases upr 95% CI	Mean outbreaks (range)	Mean VAPP	Mean DALYS	Mean total costs ($)	Total costs ($) lwr 95% CI	Total costs ($) upr 95% CI
25	Annual pSIA	156	129	183	4 (0—8)	40	2742	25173474	15992148	31700148
30	Annual pSIA	20	16	24	2 (0—6)	46	930	23728011	17672577	30762577
35	Annual pSIA	3	3	4	1 (0—5)	53	784	22400359	19353007	29825007
40	Annual pSIA	1	1	1	1 (0—3)	59	843	22627798	21033436	28887436
45	Annual pSIA	0	0	0	0 (0—3)	66	931	23512356	22713866	27949866
50	Annual pSIA	0	0	0	0 (0—3)	73	1024	24954547	24394295	29630295
55	Annual pSIA	0	0	0	0 (0—2)	80	1118	26365323	26074725	28692725
60	Annual pSIA	0	0	0	0 (0—2)	87	1214	27959358	27755154	30373154
65	Annual pSIA	0	0	0	0 (0—2)	93	1310	29595282	29435584	32053584
70	Annual pSIA	0	0	0	0 (0—2)	100	1406	31239059	31116013	33734013
75	Annual pSIA	0	0	0	0 (0—2)	107	1502	32919489	32796443	35414443
80	Annual pSIA	0	0	0	0 (0—2)	114	1598	34544941	34476873	34542323
85	Annual pSIA	0	0	0	0 (0—1)	121	1694	36207044	36157302	36157302
90	Annual pSIA	0	0	0	0 (0—1)	128	1790	37858676	37837732	37837732
95	Annual pSIA	0	0	0	0 (0—1)	135	1886	39520779	39518161	39518161
100	Annual pSIA	0	0	0	0 (0—0)	142	1983	41198591	41198591	41198591
25	Biennial pSIA	5520	5351	5689	6 (0—10)	37	77800	28308100	23428148	33900148
30	Biennial pSIA	2718	2577	2859	6 (0—10)	44	38663	30355049	22490577	35580577
35	Biennial pSIA	866	797	935	5 (0—9)	50	12822	30613905	16317007	34643007
40	Biennial pSIA	154	137	171	4 (0—8)	56	2936	29262690	17997436	33705436
45	Biennial pSIA	16	15	18	3 (0—7)	62	1093	26707196	19677866	32767866
50	Biennial pSIA	3	2	3	1 (0—5)	67	981	24900449	21358295	31830295
55	Biennial pSIA	1	1	1	1 (0—4)	73	1035	24489097	23038725	30892725
60	Biennial pSIA	0	0	0	0 (0—4)	79	1116	25446958	24719154	29955154
65	Biennial pSIA	0	0	0	0 (0—2)	86	1203	26818464	26399584	29017584
70	Biennial pSIA	0	0	0	0 (0—2)	92	1290	28305161	28080013	30698013
75	Biennial pSIA	0	0	0	0 (0—2)	98	1379	29927995	29760443	32378443
80	Biennial pSIA	0	0	0	0 (0—2)	105	1468	31556065	31440873	34058873
85	Biennial pSIA	0	0	0	0 (0—2)	111	1557	33191988	33121302	35739302
90	Biennial pSIA	0	0	0	0 (0—1)	118	1645	34835766	34801732	34801732
95	Biennial pSIA	0	0	0	0 (0—1)	124	1735	36495251	36482161	36482161
100	Biennial pSIA	0	0	0	0 (0—0)	130	1824	38162591	38162591	38162591
25	Baseline	22158	22071	22246	4 (2—9)	31	310649	20023450	16256148	29346148
30	Baseline	19021	18938	19105	4 (3—10)	37	266811	21510147	17936577	28474027
35	Baseline	15753	15664	15842	4 (3—8)	42	221134	23381691	19617007	30089007
40	Baseline	12296	12182	12409	5 (0—9)	48	172812	25499326	21297436	31769436
45	Baseline	7990	7859	8120	5 (0—9)	54	112616	28753174	25595866	33449866
50	Baseline	3524	3399	3649	6 (0—10)	60	50181	32067235	27276295	37748295
55	Baseline	598	546	650	5 (0—8)	66	9290	32310383	18484725	36810725
60	Baseline	30	26	34	3 (0—7)	70	1399	28568934	20165154	35873154
65	Baseline	2	2	2	1 (0—5)	74	1073	24979330	21845584	29699584
70	Baseline	0	0	0	0 (0—3)	80	1119	24431841	23526013	28762013
75	Baseline	0	0	0	0 (0—3)	85	1193	25612233	25206443	27824443
80	Baseline	0	0	0	0 (0—2)	91	1270	27104167	26886873	29504873
85	Baseline	0	0	0	0 (0—2)	96	1349	28685112	28567302	31185302
90	Baseline	0	0	0	0 (0—1)	102	1428	30302710	30247732	30247732
95	Baseline	0	0	0	0 (0—2)	108	1507	31964813	31928161	31928161
100	Baseline	0	0	0	0 (0—1)	113	1586	33611209	33608591	33608591

Appendix Table 7. Combined health system and GPEI perspective: table of cases, VAPP, DALYS and costs across all strategies. Uncertainty in cases and associated costs is presented as 95% credible intervals (CI). Here, the mean values are obtained from taking the mean across all model simulations, not just amongst the simulations with an outbreak (as was done in table 2 in the main text).

RI (%)	Strategy	Mean cases	Cases lwr 95% CI	Cases upr 95% CI	Mean outbreaks (range)	Mean VAPP	Mean DALYS	Mean total costs ($)	Total costs ($) lwr 95% CI	Total costs ($) upr 95% CI
25	Annual pSIA	156	129	183	4 (0—8)	40	2742	33239028	23947537	40236483
30	Annual pSIA	20	16	24	2 (0—6)	46	930	33288629	27218467	40387381
35	Annual pSIA	3	3	4	1 (0—5)	53	784	33539389	30489397	40966777
40	Annual pSIA	1	1	1	1 (0—3)	59	843	35355485	33760326	41617075
45	Annual pSIA	0	0	0	0 (0—3)	66	931	37830128	37031257	42269292
50	Annual pSIA	0	0	0	0 (0—3)	73	1024	40862666	40302187	45539473
55	Annual pSIA	0	0	0	0 (0—2)	80	1118	43863829	43573117	46192137
60	Annual pSIA	0	0	0	0 (0—2)	87	1214	47048326	46844047	49462985
65	Annual pSIA	0	0	0	0 (0—2)	93	1310	50274730	50114978	52733854
70	Annual pSIA	0	0	0	0 (0—2)	100	1406	53508993	53385908	56004730
75	Annual pSIA	0	0	0	0 (0—2)	107	1502	56779923	56656838	59275664
80	Annual pSIA	0	0	0	0 (0—2)	114	1598	59995859	59927768	59993922
85	Annual pSIA	0	0	0	0 (0—1)	121	1694	63248457	63198699	63198701
90	Annual pSIA	0	0	0	0 (0—1)	128	1790	66490579	66469629	66469630
95	Annual pSIA	0	0	0	0 (0—1)	135	1886	69743179	69740559	69740560
100	Annual pSIA	0	0	0	0 (0—0)	142	1983	73011519	73011519	73011519
25	Biennial pSIA	5520	5351	5689	6 (0—10)	37	77800	40126535	34251252	45144180
30	Biennial pSIA	2718	2577	2859	6 (0—10)	44	38663	41802331	32529975	46294381
35	Biennial pSIA	866	797	935	5 (0—9)	50	12822	42354854	27450074	47107075
40	Biennial pSIA	154	137	171	4 (0—8)	56	2936	42095010	30720649	47450549
45	Biennial pSIA	16	15	18	3 (0—7)	62	1093	41033036	33991225	47139598
50	Biennial pSIA	3	2	3	1 (0—5)	67	981	40806378	37261800	47739817
55	Biennial pSIA	1	1	1	1 (0—4)	73	1035	41983451	40532376	48388857
60	Biennial pSIA	0	0	0	0 (0—4)	79	1116	44531058	43802952	49040867
65	Biennial pSIA	0	0	0	0 (0—2)	86	1203	47492573	47073528	49692577
70	Biennial pSIA	0	0	0	0 (0—2)	92	1290	50569337	50344104	52963070
75	Biennial pSIA	0	0	0	0 (0—2)	98	1379	53782294	53614680	56233601
80	Biennial pSIA	0	0	0	0 (0—2)	105	1468	57000487	56885257	59504114
85	Biennial pSIA	0	0	0	0 (0—2)	111	1557	60226543	60155833	62774635
90	Biennial pSIA	0	0	0	0 (0—1)	118	1645	63460454	63426409	63426410
95	Biennial pSIA	0	0	0	0 (0—1)	124	1735	66710080	66696985	66696986
100	Biennial pSIA	0	0	0	0 (0—0)	130	1824	69967582	69967582	69967582
25	Baseline	22158	22071	22246	4 (2—9)	31	310649	43484360	38992552	52597540
30	Baseline	19021	18938	19105	4 (3—10)	37	266811	44364837	40580950	51953897
35	Baseline	15753	15664	15842	4 (3—8)	42	221134	45538197	42218881	52444894
40	Baseline	12296	12182	12409	5 (0—9)	48	172812	46825449	44354110	51747400
45	Baseline	7990	7859	8120	5 (0—9)	54	112616	48655160	46132766	53020230
50	Baseline	3524	3399	3649	6 (0—10)	60	50181	50433183	45595353	54122545
55	Baseline	598	546	650	5 (0—8)	66	9290	50217470	35970918	55186699
60	Baseline	30	26	34	3 (0—7)	70	1399	47667172	39241001	54975934
65	Baseline	2	2	2	1 (0—5)	74	1073	45646932	42511085	50379689
70	Baseline	0	0	0	0 (0—3)	80	1119	46687426	45781168	51019444
75	Baseline	0	0	0	0 (0—3)	85	1193	49457212	49051251	51670341
80	Baseline	0	0	0	0 (0—2)	91	1270	52538712	52321335	54940346
85	Baseline	0	0	0	0 (0—2)	96	1349	55709272	55591418	58210352
90	Baseline	0	0	0	0 (0—1)	102	1428	58916499	58861502	58861502
95	Baseline	0	0	0	0 (0—2)	108	1507	62168248	62131585	62131585
100	Baseline	0	0	0	0 (0—1)	113	1586	65404287	65401669	65401669

Incremental costs per outbreak averted

Incremental costs per outbreak averted capture the global perspective of polio eradication as a single outbreak under any vaccination strategy has implications for global polio eradication. It is important to note that the figure below does not suggest that the baseline strategy is better performing at low RI coverage levels. Instead, negative (or no) outbreaks averted by both pSIA strategies at low levels of RI coverage correspond to two phenomena: (i) the baseline strategy has larger explosive outbreaks when RI coverage is low, so the susceptible population is depleted quicker, whilst the pSIA strategies have much smaller, albeit more frequent, outbreaks when RI coverage is low.

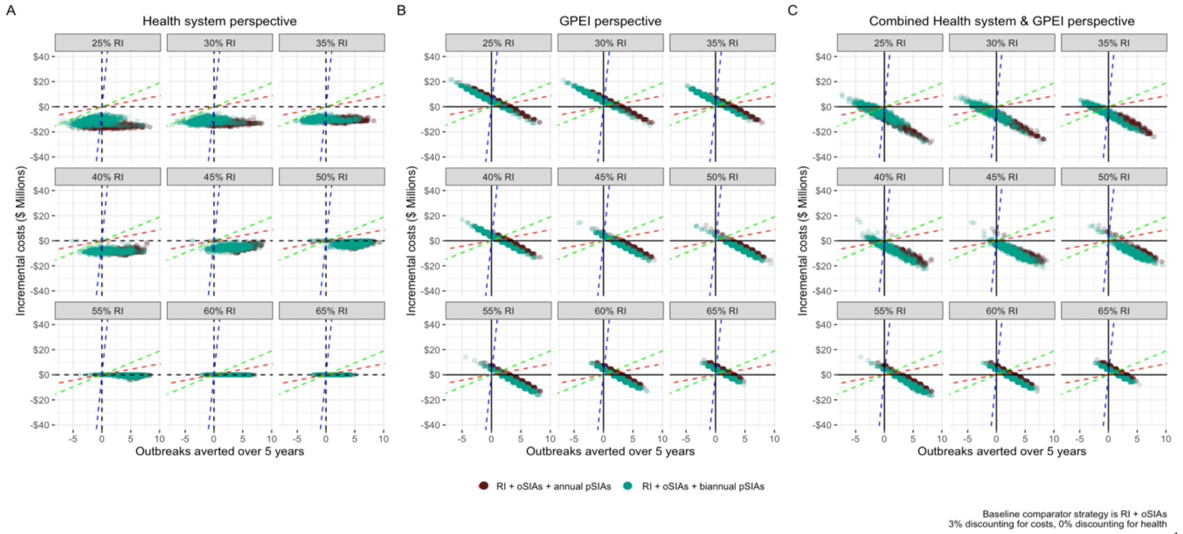

*Appendix Figure 5. Incremental costs per outbreak averted***.** *Incremental costs and outbreak averted under the annual pSIA (RI+oSIAs+annual pSIAs) and biennial pSIA (RI+oSIAs+Biennial pSIAs) strategies in comparison to the baseline strategy (RI+oSIAs) assuming a 3% discount rate for costs and 0% discount rate for health. The points correspond to 10,000 model simulations. Here, it is assumed that the cost per child in an oSIA is two times the cost per child in a pSIA. An outbreak is defined as at least one case of paralytic polio. It is important to note that this figure does not suggest that the RI+oSIAs strategy is better performing at low RI coverage levels. Instead, negative (or no) outbreaks averted by both pSIA strategies at low levels of RI coverage correspond to two phenomena: (i) the RI+oSIA strategy has larger explosive outbreaks when RI coverage is low, so the susceptible population is depleted quicker, whilst the pSIA strategies have much smaller, albeit more frequent, outbreaks when RI coverage is low.*

Section 4: Sensitivity analyses

Sensitivity analysis—discounting for health

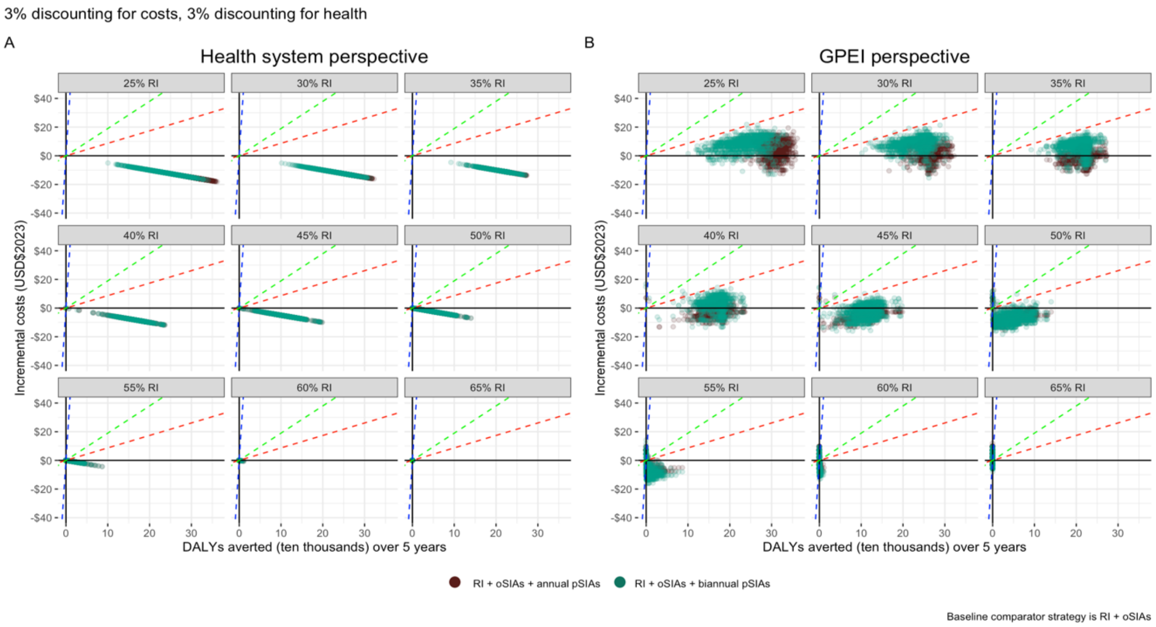

Appendix Figure 6. Incremental costs per DALY averted under the annual pSIA and biennial pSIA strategies in comparison to the baseline strategy assuming a 3% discount rate for costs and 3% discount rate for health. Incremental costs are split between health system/non-GPEI perspective and GPEI perspective and the plots are facetted by RI coverage level. Each individual model simulation is represented as a single dot. The dashed lines represent three cost-effectiveness thresholds (representing the lowest (red=Democratic Republic of the Congo), median (green=Benin) and highest (blue=South Africa) country thresholds) among low, lower middle and upper middle-income sub-Saharan African countries.

Sensitivity analysis – different assumptions about vaccine wastage

GPEI estimates OPV wastage ranges from 5-15% in SIAs and wastage is lower in SIAs than in RI [13]. Estimates of IPV wastage vary between 5-20% in the literature [14] and country-specific analyses from Nigeria and The Gambia suggest IPV wastage can even be higher in practice, exceeding 20% for IPV wastage in some instances [19, 20]. Accordingly, the main analysis assumes an average of 10% wastage for OPV in SIAs, 13% for OPV in RI and 13% for IPV.

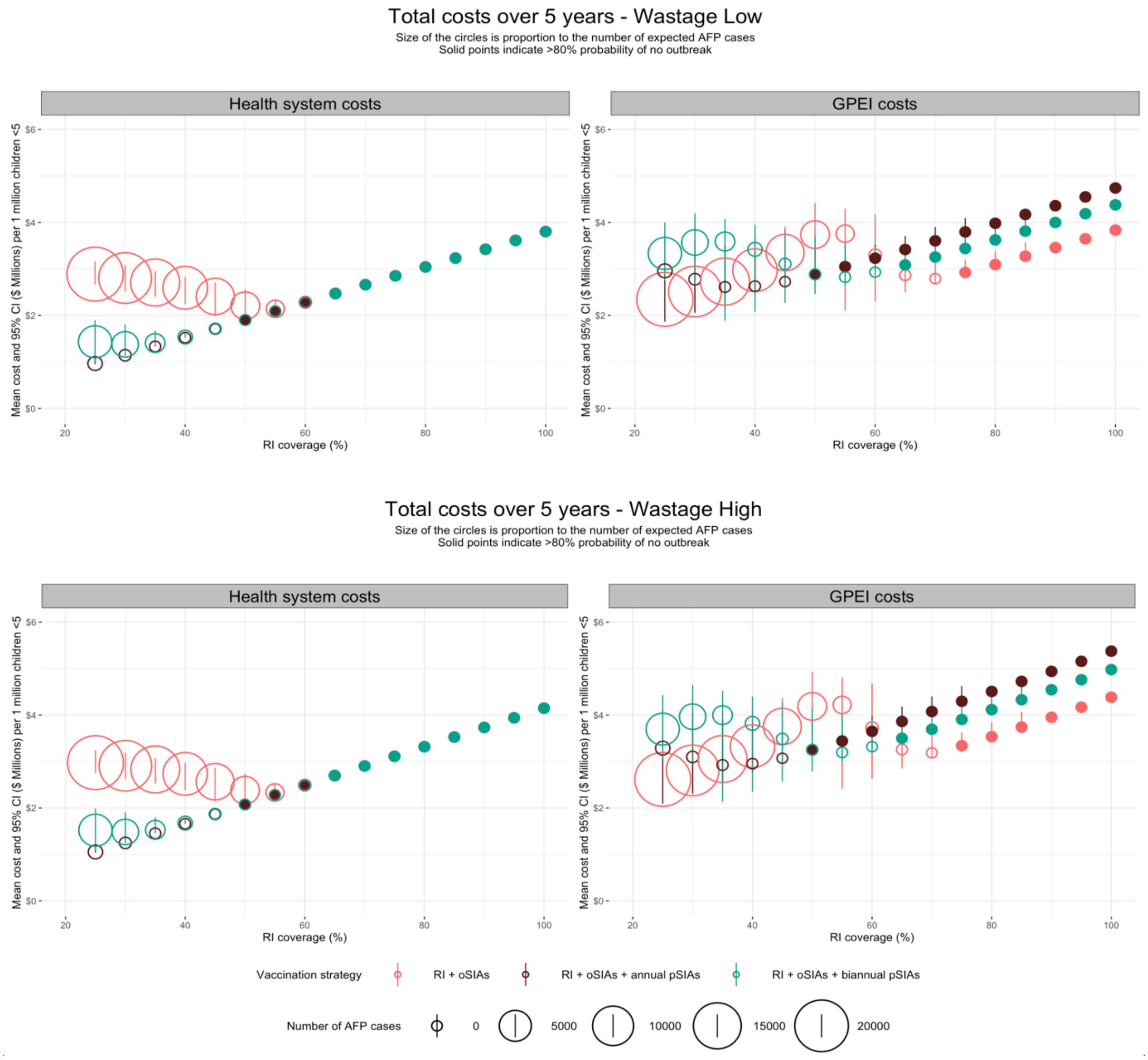

Appendix Figure 7. Total costs over five years under different wastage assumptions. The top plots represent conservative estimates of wastage using the lowest limit of the range of published wastage rates for both OPV and IPV vaccination via RI and SIAs while the bottom plots use the highest limit of the range of published wastage rates.

Sensitivity analysis – different proportional costs between pSIAs and oSIAs

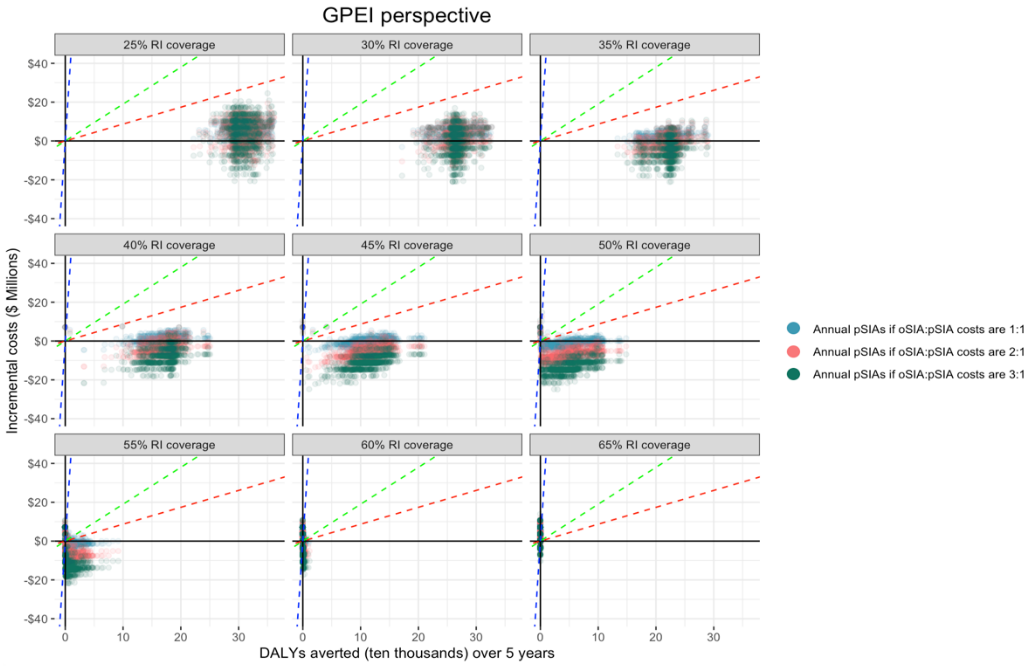

Appendix Figure 8. Sensitivity analysis exploring incremental costs per DALY averted under variable assumptions about oSIA costs. The different colours represent different proportional differences between annual pSIAs and oSIAs. For example, "Annual pSIAs if oSIA:pSIA costs are 3:1" presents the incremental costs and DALYs averted by annual pSIAs if oSIAs cost three times as much as pSIAs. Each individual model simulation is represented as a single dot. The dashed lines represent three cost-effectiveness thresholds (representing the lowest (red=Democratic Republic of the Congo), median (green=Benin) and highest (blue=South Africa) country thresholds) among low, lower middle and upper middle-income sub-Saharan African countries.

A sensitivity analysis explored incremental costs under variable assumptions about the proportional difference in costs between oSIAs and annual pSIAs. The main analysis assumed the cost per child for an oSIA was twice the cost per child for a pSIA, but available outbreak response cost data suggests a range across African countries. In the sensitivity analysis, two alternative cost assumptions were explored: (I) the cost per child in an oSIA equalled the cost per child in a pSIA (1:1) and (II) the cost per child of an oSIA was three times the cost per child in a pSIA (3:1). This sensitivity analysis answers the question, under what cost assumptions are annual pSIAs more cost-effective than oSIAs? Appendix Figure 8 shows the incremental costs per DALY averted across all oSIA assumptions and Appendix Figure 9 shows incremental costs per outbreak averted from the GPEI perspective as GPEI costs include SIA costs. When RI coverage exceeds the herd immunity threshold, the number of outbreaks, and consequently, the number of oSIAs required substantially decreases causing the GPEI incremental costs to be aligned across all oSIA cost assumptions.

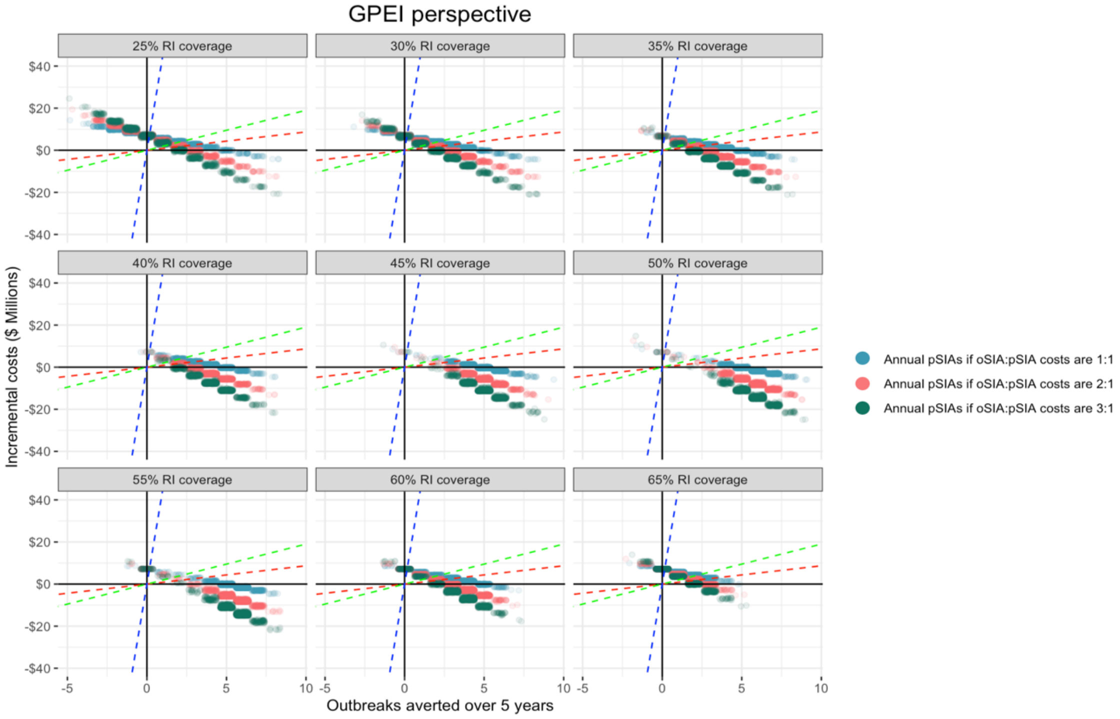

Appendix Figure 9. Sensitivity analysis exploring incremental costs per outbreak averted under variable assumptions about oSIA costs. The different colours represent different proportional differences between pSIAs and oSIAs. For example, "Annual pSIAs if oSIA:pSIA costs are 3:1" presents the incremental costs and outbreaks averted by annual pSIAs if oSIAs cost three times as much as pSIAs. Each individual model simulation is represented as a single dot. The dashed lines represent three cost-effectiveness thresholds (representing the lowest (red=Democratic Republic of the Congo), median (green=Benin) and highest (blue=South Africa) country thresholds) among low, lower middle and upper middle-income sub-Saharan African countries.

Sensitivity analysis – different assumptions about proportion of children reached by SIAs

The true effectiveness of SIAs (defined as the product of coverage and vaccine efficacy) remains uncertain in practice; although vaccine efficacy is well described in clinical trials, it is known to vary by population, and the population coverage achieved is uncertain resulting in variable effectiveness. For example, mathematical modelling demonstrates a dramatic range of SIA effectiveness estimates in Tajikistan versus the Republic of Congo: in Tajikistan, SIA effectiveness in response to a localised outbreak was estimated to be 69% (95% CI 55-80%) while in the Republic of Congo, SIA effectiveness was estimated to be 0.4% (95% CI 0.0-14.0%) per SIA [21]. In this analysis we define the SIA target population as children missed by RI. As research suggests that SIA effectiveness is highly variable in different locations, this model assessed outcomes under the assumption that all pSIAs and oSIAs reach 25% of the population missed by RI and bOPV vaccine efficacy is 50%. Historically with cVDPV outbreaks, outbreak response has occurred in a smaller, more concentrated geographical area, but in response to a WPV1 importation (as we saw in Malawi and Mozambique), the outbreak response was conducted at a national level. Therefore, equal assumptions about coverage for oSIAs and pSIAs was assumed for WPV1 outbreaks. In the sensitivity analysis below (Appendix Table 8), we explore the average number of expected AFP cases over five years and the probability of an outbreak if SIAs reached 50% of the target population, or 50% of children under five years of age who were missed by RI.

*Appendix Table 8. Expected average number of AFP cases and expected probability of an outbreak if each SIA were expected to reach 50% of the target population instead of the 25% assumption in the main analysis. Assumptions about vaccine efficacy are the same across all models. Expected AFP cases were calculated by taking the average number of AFP cases across all stochastic model simulations that had **at least one AFP case.***

RI coverage	SIA strategy	If SIAs vaccinate 25% of the target population		If SIAs vaccinate 50% of the target population	
		Average number of expected AFP cases over 5 years	Expected probability of an outbreak	Average number of expected AFP cases over 5 years	Expected probability of an outbreak
25%	RI + oSIA + annual pSIA	176	88%	1	16%
35%	RI + oSIA + annual pSIA	5	65%	1	9%
50%	RI + oSIA + annual pSIA	1	20%	1	6%
75%	RI + oSIA + annual pSIA	1	4%	1	2%
25%	RI + oSIA + biennial pSIA	5,564	99%	15	83%
35%	RI + oSIA + biennial pSIA	894	97%	3	56%
50%	RI + oSIA + biennial pSIA	4	67%	1	17%
75%	RI + oSIA + biennial pSIA	1	6%	1	6%
25%	RI + oSIA	22,158	100%	18,093	100%
35%	RI + oSIA	15,753	100%	9,851	100%
50%	RI + oSIA	3,549	99%	602	99%
75%	RI + oSIA	1	14%	1	14%

Sensitivity analysis – different R_0 assumptions

As the true value of R_0 for polio is unknown and depends on geographical settings, sanitation and hygiene, and age. In the main analysis, we assume an R_0 of 3, which is in line with other research and the target population of children under five years of age living in an LMIC in Africa. However, in the sensitivity analysis below (Appendix Table 9) we explore the average number of expected AFP cases over five years and the probability of an outbreak if R_0 = 6. As shown the table, when RI coverage >50%, a higher R_0 only increases the probability of an outbreak slightly in the annual pSIA strategy but has a bigger effect on outbreak probability for the biennial pSIA and baseline strategies.

*Appendix Table 9. Expected number of AFP cases and expected probability of an outbreak under different R_0 assumptions. Expected AFP cases were calculated by taking the average number of AFP cases across all stochastic model simulations that had **at least one AFP case.***

RI coverage	SIA strategy	R_0 = 3		R_0 = 6	
		Average number of expected AFP cases over 5 years	Expected probability of an outbreak	Average number of expected AFP cases over 5 years	Expected probability of an outbreak
25%	RI + oSIA + annual pSIA	176	88%	16,379	100%
50%	RI + oSIA + annual pSIA	1	20%	3,837	100%
75%	RI + oSIA + annual pSIA	1	4%	1	19%
25%	RI + oSIA + biennial pSIA	5,564	99%	24,403	100%
50%	RI + oSIA + biennial pSIA	4	67%	9,542	100%
75%	RI + oSIA + biennial pSIA	1	6%	4	68%
25%	RI + oSIA	22,158	100%	37,069	100%
50%	RI + oSIA	3,549	99%	20,229	100%
75%	RI + oSIA	1	14%	2,046	99%

Sensitivity analysis – different importation rates

When RI coverage exceeds 50%, model assumptions for the importation rate of WPV infection have little effect on the expected number of AFP cases and expected probability of an outbreak across all vaccination strategies. This reiterates the importance of baseline RI coverage, which remains an important underlying factor that greatly influences the number of expected AFP cases and outbreak probability, more so than importation rate or assumptions around the proportion of children reached by SIAs.

Seasonality, with peaks in infection usually seen around late summer and autumn, has been observed for WPV in Northern Hemisphere endemic settings (Afghanistan and Pakistan), but remains a phenomenon not well explored for geographies in the Southern Hemisphere [22]. Furthermore, here outbreaks are dependent on WPV1 importations, which can occur at any time. To better understand if any trends in importations exist, better data on migration and travel patterns into African countries from endemic settings is needed.

Appendix Table 10. Expected number of AFP cases and expected probability of an outbreak if the rate of WPV importation is one importation of WPV infection every year and three importations every year, rates that are under and over the rate used in the main analysis (two importations per year). Expected AFP cases were calculated by taking the average number of AFP cases across all stochastic model simulations that had ***at least one AFP case.***

RI coverage	SIA strategy	1 importation every year		3 importations every year	
		Average number of expected AFP cases over 5 years	Expected probability of an outbreak	Average number of expected AFP cases over 5 years	Expected probability of an outbreak
25%	RI + oSIA + annual pSIA	42	71%	49	96%
50%	RI + oSIA + annual pSIA	1	11%	1	27%
75%	RI + oSIA + annual pSIA	1	2%	1	5%
25%	RI + oSIA + biennial pSIA	5,353	95%	5,363	100%
50%	RI + oSIA + biennial pSIA	4	45%	5	78%
75%	RI + oSIA + biennial pSIA	1	3%	1	8%
25%	RI + oSIA	22,327	99%	22,236	100%
50%	RI + oSIA	3,430	91%	3,399	100%
75%	RI + oSIA	1	9%	1	20%

Section 5: Supporting information

Raw data used to inform proportional differences between pSIA and oSIA costs

*Appendix Table 11. Country level SIA data showing the differences in costs between oSIAs and pSIAs. The rightmost column shows the proportional difference between oSIAs and pSIAs for operational costs. *pSIA cost data was available pre COVID-19 pandemic and given in USD$2019, therefore, estimates have been calculated for USD$2023 assuming 2019$1 = 2023$1.18. oSIA cost data for bOPV has historically been less readily available. Therefore, to estimate the proportional differences between oSIAs and pSIAs, oSIA cost data from campaigns administering novel OPV2 (nOPV2) was used, and we assume that while the vaccine costs may differ, the oSIA operational costs are similar.*

Country	bOPV cost (USD$)	pSIA - cost per child $2019	pSIA - cost per child $2023*	oSIA (nOPV) - cost per child $2023	proportional difference - *operational only*
Benin	0.17	0.31	0.37	0.73	2.0
Burkina Faso	0.15	0.27	0.32	0.65	2.0
Cameroon	0.17	0.33	0.39	0.86	2.2
Central African Republic	0.17	1.09	1.29	2.79	2.2
Chad	0.16	0.45	0.53	0.42	0.8
Congo	0.16	0.44	0.52	2.29	4.4
Côte d'Ivoire	0.14	0.15	0.18	0.67	3.8
DR Congo	0.15	0.48	0.57	1.28	2.3
Eritrea	0.18	0.95	1.12	1.00	0.9
Ethiopia	0.17	0.89	1.05	0.56	0.5
Gabon	0.16	0.77	0.91	1.00	1.1
Gambia	0.18	0.60	0.71	0.78	1.1
Ghana	0.18	0.29	0.34	1.32	3.9
Guinea	0.19	0.26	0.31	1.23	4.0
Kenya	0.18	0.59	0.70	1.02	1.5
Liberia	0.20	0.83	0.98	1.58	1.6
Madagascar	0.17	0.37	0.44	0.53	1.2
Malawi	-	-	0.00	1.00	
Mali	0.17	0.24	0.28	0.52	1.8
Mauritania	0.12	0.81	0.96	1.00	1.0
Mozambique	-	-	0.00	0.80	
Niger	0.19	0.36	0.42	0.57	1.3
Nigeria	0.21	0.32	0.38	0.22	0.6
Senegal	0.18	0.29	0.34	0.47	1.4
Sierra Leone	0.16	0.46	0.54	0.92	1.7
South Sudan	0.17	0.78	0.92	1.17	1.3
Sudan	0.15	0.58	0.68	0.65	0.9
Tanzania	0.17	0.93	1.10	1.00	0.9
Togo	0.17	0.27	0.32	0.73	2.3
Uganda	0.18	0.48	0.57	1.00	1.8
Zambia	-	-		2.78	
Zimbabwe	-	-		1.76	
**Average**	**$0.17**	**$0.52**	**$0.61**	**$1.04**	**$1.7**

Cost-effectiveness plane

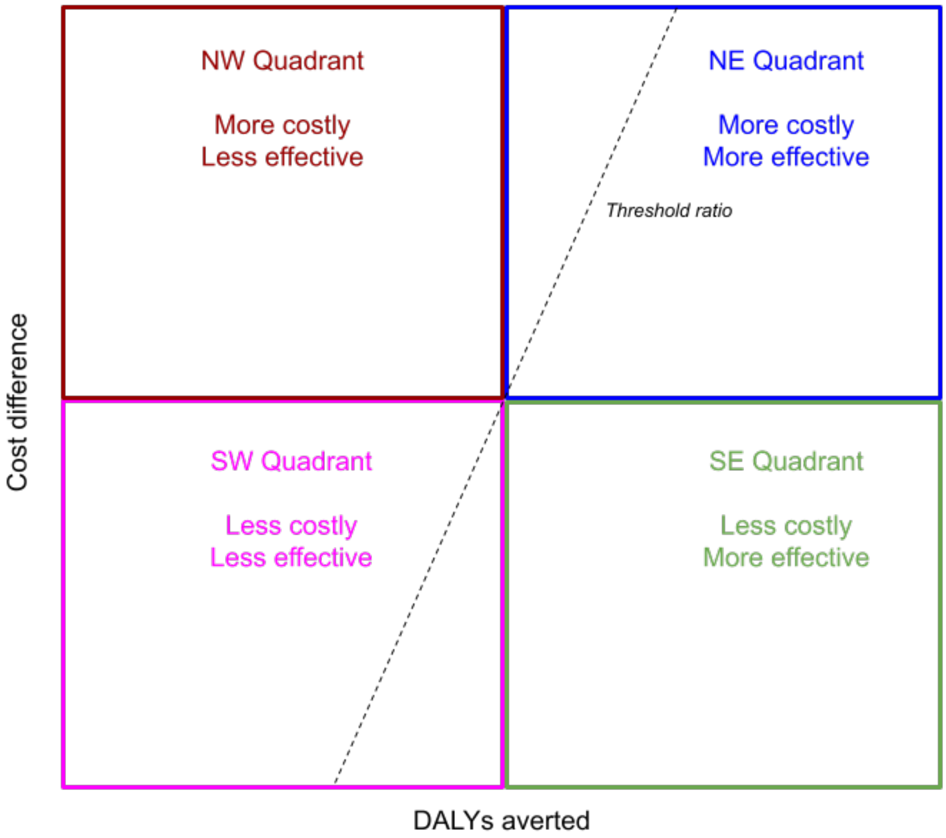

Appendix Figure 10. Quadrants comprising a cost-effectiveness plane for interpretation of incremental costs and DALYs averted under each vaccination strategy. This figure was adapted for this analysis from Briggs et al. 2006 [23]. If the ICER (cost differences / DALYS averted) for a particular vaccination strategy falls below the threshold ratio (dashed line – which represents the willingness-to-pay), then the strategy represents a cost-effective option.

Appendix Table 12. Consolidated Health Economic Evaluation Reporting Standards 2022 (CHEERS 2022) checklist. The checklist has been taken from Husereau et al. and adapted to this analysis [24].

Section/topic	Item No	Guidance for reporting	Reported in section
**Title**			
Title	1	Identify the study as an economic evaluation and specify the interventions being compared.	Heading
**Abstract**			
Abstract	2	Provide a structured summary that highlights context, key methods, results, and alternative analyses	Heading
**Introduction**			
Background and objectives	3	Give the context for the study, the study question, and its practical relevance for decision making in policy or practice.	Introduction
**Methods**			
Health economic analysis plan	4	Indicate whether a health economic analysis plan was developed and where available	Methods paragraphs 1 & 2
Study population	5	Describe characteristics of the study population (such as age range, demographics, socioeconomic, or clinical characteristics).	Methods paragraph 2
Settings and location	6	Provide relevant contextual information that may influence findings	Methods paragraph 2
Comparators	7	Describe the interventions or strategies being compared and why chosen.	Methods: "vaccination strategies"
Perspective	8	State the perspective(s) adopted by the study and why chosen.	Methods: "Perspectives"
Time horizon	9	State the time horizon for the study and why appropriate.	Methods: "Time horizon and model assumptions"
Discount rate	10	Report the discount rate(s) and reason chosen.	Methods: "Health and economic outcomes"
Selection of outcomes	11	Describe what outcomes were used as the measure(s) of benefit(s) and harm(s).	Methods: "Probability of an outbreak occurring" & "DALYS"
Measurement of outcomes	12	Describe how outcomes used to capture benefit(s) and harm(s) were measured.	Methods: "Health and economic outcomes" & "Adverse events"
Valuation of outcomes	13	Describe the population and methods used to measure and value outcomes.	Methods: "Time horizon and model assumptions"
Measurement and valuation of resources and costs	14	Describe how costs were valued.	Methods: "Vaccine costs" & Appendix section 2
Currency, price data and conversion	15	Report the dates of the estimated resource quantities and unit costs, plus the currency and year of conversion.	Methods: "Vaccine costs"
Rationale and description of model	16	If modelling is used, describe in detail and why used. Report if the model is publicly available and where it can be accessed.	Methods: "Model structure" & Appendix sections 1 & 2
Analytics and assumptions	17	Describe any methods for analysing or statistically transforming data, any extrapolation methods, and approaches for validating any model used.	Methods: "Time horizon and model assumptions" & Appendix sections 1 & 2

Characterising heterogeneity	18	Describe any methods used for estimating how the results of the study vary for subgroups.	We assume a homogenous population
Characterising distributional effects	19	Describe how impacts are distributed across different individuals or adjustments made to reflect priority populations.	We assume SIAs reach hard-to-reach children otherwise missed by RI
Characterising uncertainty	20	Describe methods to characterise any sources of uncertainty in the analysis.	Appendix section 4
Approach to engagement with patients and others affected by the study	21	Describe any approaches to engage patients or service recipients, the general public, communities, or stakeholders (such as clinicians or payers) in the design of the study.	GPEI stakeholders were involved in the analysis from project inception
**Results**			
Study parameters	22	Report all analytic inputs (such as values, ranges, references) including uncertainty or distributional assumptions.	Results table 2
Summary of main results	23	Report the mean values for the main categories of costs and outcomes of interest and summarise them in the most appropriate overall measure.	Results paragraph 1
Effect of uncertainty	24	Describe how uncertainty about analytic judgments, inputs, or projections affect findings. Report the effect of choice of discount rate and time horizon, if applicable.	Appendix section 4
Effect of engagement with patients and others affected by the study	25	Report on any difference patient/service recipient, general public, community, or stakeholder involvement made to the approach or findings of the study	This project has been presented at conferences and stakeholder meetings and was well received
**Discussion**			
Study findings, limitations, generalisability, and current knowledge	26	Report key findings, limitations, ethical or equity considerations not captured, and how these could affect patients, policy, or practice.	Discussion
**Other relevant information**			
Source of funding	27	Describe how the study was funded and any role of the funder in the identification, design, conduct, and reporting of the analysis.	Acknowledgements
Conflicts of interest	28	Report authors conflicts of interest according to journal or International Committee of Medical Journal Editors requirements.	Declaration of Interests

References

1. Bandyopadhyay AS, Modlin JF, Wenger J, Gast C. Immunogenicity of New Primary Immunization Schedules With Inactivated Poliovirus Vaccine and Bivalent Oral Polio Vaccine for the Polio Endgame: A Review. Clin Infect Dis. 2018;67(suppl_1):S35-s41. Epub 2018/10/31. doi: 10.1093/cid/ciy633. PubMed PMID: 30376081; PubMed Central PMCID: PMCPMC6206125.
2. Gao H, Lau EHY, Cowling BJ. Waning Immunity After Receipt of Pertussis, Diphtheria, Tetanus, and Polio-Related Vaccines: A Systematic Review and Meta-analysis. The Journal of Infectious Diseases. 2021;225(4):557-66. doi: 10.1093/infdis/jiab480.
3. Fine PEM, Carneiro IAM. Transmissibility and Persistence of Oral Polio Vaccine Viruses: Implications for the Global Poliomyelitis Eradication Initiative. American Journal of Epidemiology. 1999;150(10):1001-21. doi: 10.1093/oxfordjournals.aje.a009924.
4. Nathanson N, Kew OM. From emergence to eradication: the epidemiology of poliomyelitis deconstructed. American journal of epidemiology. 2010;172(11):1213-29. Epub 2010/10/26. doi: 10.1093/aje/kwq320. PubMed PMID: 20978089.
5. Kalkowska DA, Badizadegan K, Thompson KM. Outbreak management strategies for cocirculation of multiple poliovirus types. Vaccine. 2023.
6. United Nations. World Population Prospects 2022 2022. Available from: https://population.un.org/wpp/.
7. Utazi CE, Thorley J, Alegana VA, Ferrari MJ, Takahashi S, Metcalf CJE, et al. Mapping vaccination coverage to explore the effects of delivery mechanisms and inform vaccination strategies. Nat Commun. 2019;10(1):1633. Epub 2019/04/11. doi: 10.1038/s41467-019-09611-1. PubMed PMID: 30967543; PubMed Central PMCID: PMCPMC6456602.
8. Zimmermann M, Hagedorn B, Lyons H. Projection of Costs of Polio Eradication Compared to Permanent Control. The Journal of Infectious Diseases. 2019;221(4):561-5. doi: 10.1093/infdis/jiz488.
9. Portnoy A, Ozawa S, Grewal S, Norman BA, Rajgopal J, Gorham KM, et al. Costs of vaccine programs across 94 low-and middle-income countries. Vaccine. 2015;33:A99-A108.
10. UNICEF. Inactivated Polio Vaccine (IPV) price data [26 April 2023]. Available from: https://www.unicef.org/supply/media/16266/file/IPV-vaccine-prices-14032023.pdf.
11. Kalkowska DA, Thompson KM. Health and economic outcomes associated with polio vaccine policy options: 2019–2029. Risk Analysis. 2021;41(2):364-75.
12. Ni L, Seward JF, Santibanez TA, Pallansch MA, Kew OM, Prevots DR, et al. Vaccine policy changes and epidemiology of poliomyelitis in the United States. Jama. 2004;292(14):1696-701.
13. UNICEF. Guidance Note on Cold Chain Logistics and Vaccine Management During Polio Supplementary Immunization Activities. UNICEF, New York, NY; 2015.
14. Mvundura M, Hsu J-S, Frivold C, Kristensen D, Boyle S, Zehrung D, et al. Evaluating the cost per child vaccinated with full versus fractional-dose inactivated poliovirus vaccine. Vaccine: X. 2019;2:100032.
15. Pichon-Riviere A, Drummond M, Palacios A, Garcia-Marti S, Augustovski F. Determining the efficiency path to universal health coverage: cost-effectiveness thresholds for 174 countries based on growth in life expectancy and health expenditures. The Lancet Global Health. 2023;11(6):e833-e42.
16. World Bank. GDP (current US$) - Sub-Saharan Africa. 2022.
17. Global Polio Eradication Initiative. Fact sheet: vaccine-associated paralytic polio (VAPP) and vaccine-derived poliovirus (VDPV) 2015. Available from: https://www.mchodessa.com/documents/systems/blog/VAPPandcVDPVFactSheet-Feb2015.pdf.
18. Tebbens RJD, Pallansch MA, Kew OM, Cáceres VM, Jafari H, Cochi SL, et al. Risks of paralytic disease due to wild or vaccine-derived poliovirus after eradication. Risk Analysis. 2006;26(6):1471-505.
19. Wallace AS, Willis F, Nwaze E, Dieng B, Sipilanyambe N, Daniels D, et al. Vaccine wastage in Nigeria: An assessment of wastage rates and related vaccinator knowledge, attitudes and practices.

Vaccine. 2017;35(48 Pt B):6751-8. Epub 2017/10/27. doi: 10.1016/j.vaccine.2017.09.082. PubMed PMID: 29066189; PubMed Central PMCID: PMCPMC5771486.
20. Thompson KM, Kalkowska DA. Potential future use, costs, and value of poliovirus vaccines. Risk Analysis. 2021;41(2):349-63.
21. Blake IM, Martin R, Goel A, Khetsuriani N, Everts J, Wolff C, et al. The role of older children and adults in wild poliovirus transmission. Proceedings of the National Academy of Sciences. 2014;111(29):10604-9.
22. Martinez-Bakker M, King AA, Rohani P. Unraveling the Transmission Ecology of Polio. PLoS Biol. 2015;13(6):e1002172. Epub 2015/06/20. doi: 10.1371/journal.pbio.1002172. PubMed PMID: 26090784; PubMed Central PMCID: PMCPMC4474890.
23. Briggs A, Sculpher M, Claxton K. Decision modelling for health economic evaluation: Oup Oxford; 2006.
24. Husereau D, Drummond M, Augustovski F, de Bekker-Grob E, Briggs AH, Carswell C, et al. Consolidated Health Economic Evaluation Reporting Standards 2022 (CHEERS 2022) statement: updated reporting guidance for health economic evaluations. BMJ. 2022;376:e067975. doi: 10.1136/bmj-2021-067975.
